# Automation of flow cytometry data analysis with elastic image registration

**DOI:** 10.1038/s41598-025-99118-1

**Published:** 2025-05-15

**Authors:** Allison Irvine, Mohamed Mahmoud Moustafa, Sahul Patel, Aniket Patel, Lilja Hardardottir, Francesca Delvecchio, Taylor Foreman, Jean Oak, Scott J. Bornheimer, Raffaello Cimbro

**Affiliations:** 1 BD Biosciences, Milpitas, CA USA; 2 Dynamic Omics, Centre for Genomics Research, Discovery Sciences, R&D, AstraZeneca, Gaithersburg, MD USA; 3 Cell and Gene Therapy Manufacturing, Novartis, Morris Plains, NJ USA; 4 Dynamic Omics, Center for Genomics Research, Discovery Sciences, R&D, AstraZeneca, Cambridge, UK; 5 Oncology IO, R&D, AstraZeneca, Cambridge, UK; 6 Oncology, Immune Engagers, R&D, AstraZeneca, Gaithersburg, MD USA; 7 Department of Clinical Pathology, Stanford University School of Medicine, Stanford, CA USA

**Keywords:** Flow cytometry, Immunology, Computational biology and bioinformatics, Image processing

## Abstract

Cell populations in flow cytometry are typically identified via visual manual gating, a time-consuming and error-prone approach to select subpopulations based on expression of cellular markers. Batch processing can be used to automate the analysis of bimodally distributed data but underperforms with highly-variable or continuously-expressed markers. We developed a visual pattern recognition automated gating tool, BD ElastiGate Software (hereafter ElastiGate), to recapitulate the visual process of manual gating by automatically adjusting gates to capture local variability. ElastiGate converts histograms and two-dimensional plots into images, then uses elastic B-spline image registration to transform pre-gated training plot images and their gates to corresponding ungated target plot images, thereby adjusting for local variations. ElastiGate was validated with biologically relevant datasets in CAR-T cell manufacturing, tumor-infiltrating immunophenotyping, cytotoxicity assays (> 500 data files), and a high-dimensional dataset. Accuracy was evaluated against corresponding manually gated analysis using F1 score statistics. ElastiGate performed similarly to manual gating, with average F1 scores of > 0.9 across all gates. ElastiGate, accessible as a FlowJo Software plugin or in BD FACSuite Software, uses minimal training samples to automate gating while substantially reducing analysis time and outperforming existing 2D plot autogating solutions in F1 scores and ease of implementation.

**Supplementary Information:**

The online version contains supplementary material available at 10.1038/s41598-025-99118-1.

## Introduction

Flow cytometry is a single-cell resolution technology used to identify and quantify cell subpopulations and can simultaneously characterize the expression of more than 40 cell surface and intracellular markers at a rate of several thousand cells per second. Common applications include drug development, immunophenotyping, cell therapy manufacturing, clinical diagnostics, and high-dimensional exploratory assays to identify novel biomarkers and cell subsets. Traditionally, flow cytometry data are analyzed using bivariate or histogram plots in which gates, or boundaries around populations, are drawn manually for all plots in a gating hierarchy for each sample (data file). Once a template gating strategy has been defined, it can be batch applied to each data file. Although batch processing improves the speed of analysis, it still requires visual confirmation to ensure the correct placement of each gate for each file to account for biological or technical variability in the data. This process can be error-prone, time-consuming, and subjective, potentially introducing user variability that can impact the accuracy and repeatability of the results, especially in samples with highly variable data, continuously expressed markers, and complex gating strategies.

Automated flow cytometry analysis, both supervised and unsupervised, is a prominent area of active development^1–13^. The biggest challenge of this approach is to account for biological and experimental variability at the level of individual files and automatically adjust the local gating structure. Current approaches rely on clustering algorithms or attempts to replicate manual gating through statistical approaches such as event density, which tend to be difficult to standardize and may underperform in comparison with expert manual gating^1,2^. One of such algorithms is flowDensity, a leading tool for automating the analysis of research samples by applying pre-established gating hierarchy, using a tailored signal peak or percentile thresholds for each population^9,12^, but may require computational expertise for optimization. There is an unmet need for objective, consistent and automatic gating process using a preexisting gating template that is straightforward for the non-computational expert to use.

We developed an automated gating method called BD ElastiGate™ Software (from now on referred to as ElastiGate)^14^ that, using minimal pre-gated training data, can adapt gates to variations in new data while following the intended template gating strategy. Unlike existing clustering- or density-based approaches, this method was inspired by the observation that manual gating is inherently based on visual pattern recognition by the analyst. Flow cytometry data in two-dimensional (2D) plots and histograms are therefore converted to images. Local shifts in ungated target data, compared with training data, are modeled using elastic image registration. A transformation is determined that warps the training data plots to the corresponding new, ungated plots, and the same transformation is then applied to the gate vertices. This allows gate vertices for any gate shape to follow shifts in the nearby data and maintain gate “cutoffs” intended by the user. No assumptions are made about population shapes, and peak finding and density cutoffs are not used. The method is implemented as a plugin for the widely available FlowJo™ Software and in BD FACSuite™ Software (from now on referred to as FlowJo and FACSuite, respectively) and is designed for biologists or technicians. The generated gates can be further reviewed and modified if necessary.

To demonstrate the performance of ElastiGate, we compared gating accuracy with manual gating by multiple experts for a range of real-world flow cytometry datasets that varied in complexity and difficulty. As a comparator, for one data set, ElastiGate was also tested alongside two alternative methods, flowDensity^9,12^ and Cytobank Automatic gating, for automated gating of 2D plots. Across these biological applications, F1 score results showed that ElastiGate performed very similarly to expert manual gating. ElastiGate is a tool with the potential to improve the consistency and objectivity of analyses across samples and operators while increasing operator productivity and decreasing analysis time.

## Results

ElastiGate was developed and validated using ad hoc–designed benchmarking data, followed by extensive validation with biologically relevant datasets.

### Development benchmarking

#### Lysed whole-blood scatter gating

ElastiGate was initially evaluated for its ability to correctly identify lymphocytes, monocytes, and granulocytes in 31 blood-derived samples based on forward scatter (FSC) and side scatter (SSC) light profiles. The data set was variable owing to the red blood cell lysis sample-processing protocol, making it an ideal candidate to evaluate how the proposed method recognized and adapted to local changes. Training gates and the ElastiGate results on four representative target files are shown in Supplementary Figure 1A.

**Fig. 1:**
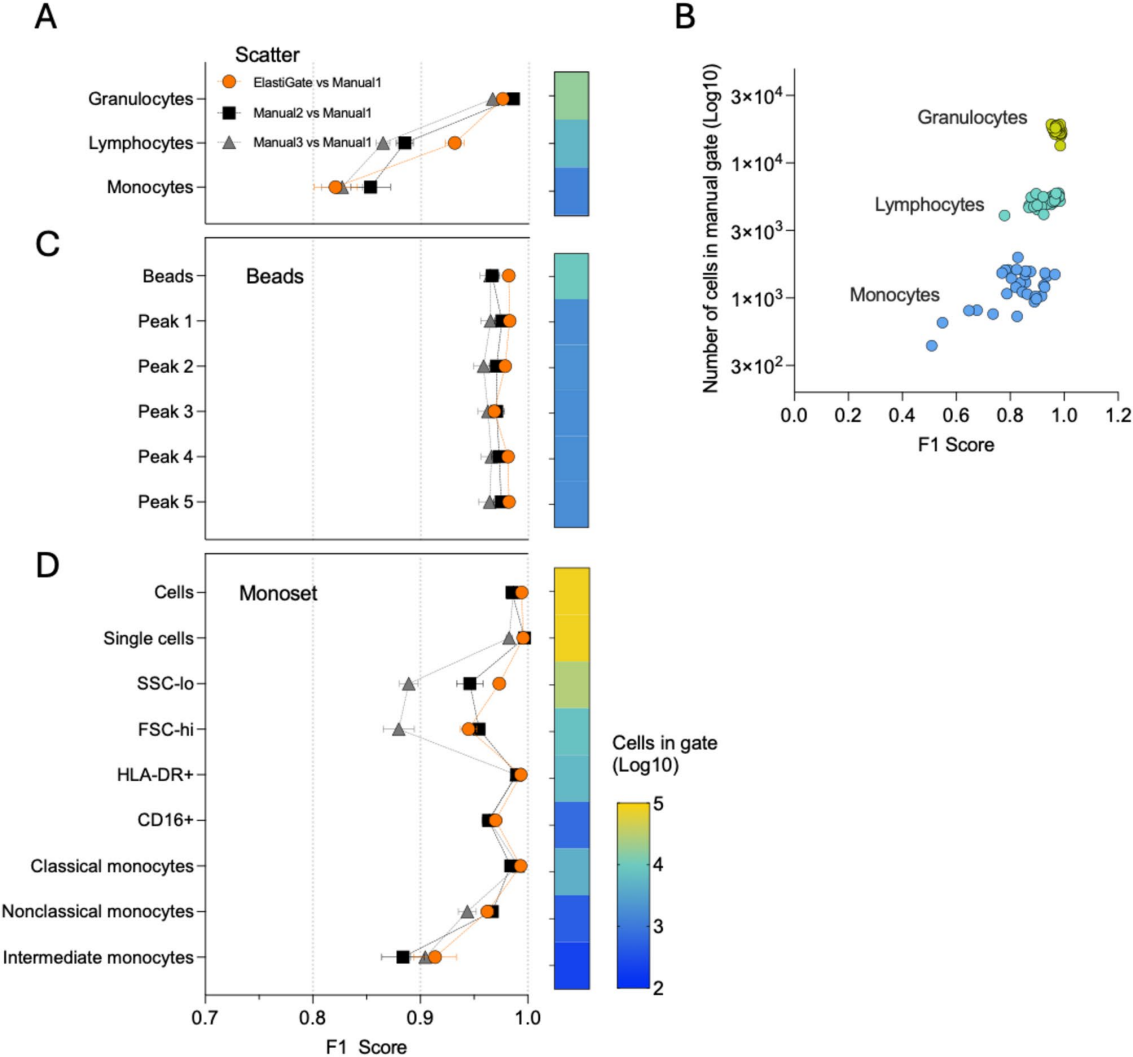
ElastiGate application on three benchmarking datasets. (**A**) The distributions of F1 scores for each gate over 30 target fcs files in the lysed whole blood scatter dataset, comparing ElastiGate to gates drawn by three manual expert analysts (Manual 1–3). Lymphocytes, Granulocytes, and Monocytes gates on FSC-A versus SSC-A plot were provided from one training file. The heatmap bar represents number of cells in each gate as a Log10 scale. Values represent the mean and error bars represent standard error of the mean (SEM). (**B**) The number of cells in each population plotted against its ElastiGate F1 score from the lysed whole blood scatter dataset. (**C**) The distributions of F1 scores for each gate over 30 target fcs files in the multilevel fluorescence quantitation beads dataset. (**D**) The distributions of F1 scores for each gate over 20 fcs files in the monocyte subset analysis dataset. ElastiGate was used with one training file.

A single manually gated sample was used as training set, ElastiGate was applied to the remaining 30 samples, and results were compared against manual gating conducted by three expert analysts (Fig. 1A). For the purposes of statistical analysis, one of the manually gated datasets was selected as ground truth. In this data set, granulocytes segregated as a defined population and lymphocytes posed more of a challenge due to the proximity of remaining red blood cells and debris, whereas monocytes showed the greatest variability driven by low event counts (Fig. 1A, B) and partial overlap with the granulocyte population. The median F1 scores for ElastiGate for the granulocyte, lymphocyte, and monocyte gates reflected the increased difficulty in defining these populations (0.979, 0.944, and 0.841, respectively). ElastiGate tracked the shifting blood-derived populations on FSC-SSC profiles with a definition almost identical to those of the two manual analyses (Fig. 1A, B).

#### Multilevel fluorescence quantitation beads

Fluorescence quantitation beads with different bead populations bound to known numbers of fluorescent molecules are routinely used to calibrate the fluorescence scale of flow cytometers and to quantify the cell surface density of target antigens. The process of gating individual bead populations can be time consuming when minor gate adjustments are necessary to account for variations in the acquisition conditions or when comparing different bead lots. The selected data set included quantitation beads used routinely in a multicolor antigen density assay for targetable markers in B-cell malignancies^15^ and was chosen to evaluate the accuracy of ElastiGate to handle multiple histogram linear gates.

A single sample was manually gated on FSC-A and SSC-A to identify the main bead population, followed by five histogram gates to identify the bead populations with different fluorescence levels. This template was used to train ElastiGate and was applied to the remaining 20 samples. The same data set was also gated by three analysts, and the result of one was selected as ground truth. Training gates and ElastiGate results on four of the target files are shown in Supplementary Figure 1B.

The performance of ElastiGate was highly analogous to that of manual gating (Fig. 1C). ElastiGate correctly identified and compensated for shifts among samples; median F1 scores were 0.991 for all the gates, and the median F1 scores of the other two analysts were 0.972 and 0.964. The lowest overall F1 score (over all gates and samples) for ElastiGate was 0.92, whereas the lowest overall F1 scores for the two analysts were 0.87 and 0.90.

#### Monocyte subset analysis

Next, we challenged ElastiGate with the analysis of monocyte populations that lack simple bimodal expression. The dataset included 20 blood samples with four fluorescence parameters, in addition to FSC and SSC, to identify classical, intermediate, and non-classical monocytes. Complexity was further increased by varying the sample processing conditions and flow rates during the acquisition.

A single sample was manually gated, used to train ElastiGate, and applied to the remaining 19 samples. Three analysts manually gated the complete data set, and one analysis was randomly selected as ground truth for statistical purposes. Training gates and ElastiGate results on representative target files are shown in Supplementary Figure 2.

ElastiGate was used to analyze all the plots except for the initial gate to remove FSC and SSC events outside the linear range that could not be properly interpreted by the mathematical model. ElastiGate was applied to nine gates over seven plots and was run with the density level set to 1 for the first five gates in the hierarchy, then set to 0 for other gates to account for the reduced number of events, as explained in the Methods and Discussion sections. The median ElastiGate F1 scores were all > 0.93 (Fig. 1D). The minimum F1 score over all gates was 0.82, with the exception of one partially undefined intermediate monocyte population, which had an F1 score of 0.597, associated with the lowest number of cells within the gate. ElastiGate median F1 scores were comparable to those of two other analysts for all the gates (F1 difference, ≤ 0.015). Of note, for the “SSC-lo” and “FSC-hi” gates, one analyst followed a gating pattern different from all the other analysts and ElastiGate. This further corroborates individual subjectivity as a source of variability in flow cytometry data analysis that can be prevented through standardized approaches.

### Validation with biologically relevant datasets

After the initial development and benchmarking, ElastiGate was validated by using three biologically relevant datasets. These datasets were selected with the additional goal of demonstrating how to achieve accuracy while greatly simplifying the workflow. The evaluation of ElastiGate included a chimeric antigen receptor (CAR)-T cell therapy quality control assay, a tumor infiltrating lymphocyte (TIL) immunophenotyping assay, and a cytotoxicity screening assay.

#### Cell therapy quality control testing

Cell therapy manufacturing often includes flow cytometry quality control (QC) testing according to standard operating procedures (SOPs), but patient variability and heterogenous expression of cell modifications require individual review of each analysis. We tested ElastiGate on 19 samples from incoming leukapheresis samples and six from final release cell therapy products of T cells transfected with a CAR targeting CD19. Each sample included accompanying isotype and fluorescence-minus-one (FMO) anti-CAR controls. In some instances, replicates were included, bringing the total number of data files to 75. Common “clean up” gates were applied to all samples, and sample-specific subsequent gates for leukapheresis and CAR-T cells were applied to the FMO and fully stained sample files.

For the leukapheresis set, three random data files were gated and used as training data for ElastiGate to adjust the first five gates (“clean up” gates) onto the remaining 54 data files. We then used the same three training files to apply ElastiGate to the remaining gates onto 19 FMO and 16 fully stained samples. We repeated a similar process for the CAR-transduced T-cell files, using one fully stained sample file as training data. In the leukapheresis set, the CAR-transduced population was expected to be close to zero. For the CAR-transduced cell files, only the fully stained sample flow cytometry standard (FCS) files had an expected number of cells of > 0 in the transduction gate. For both leukapheresis and final release product sets, the isotype files were excluded from gates after the first five “clean up” gates. The density level was set to 0 to ensure maximum sensitivity to changes in sparse areas of the plots. Training gates and ElastiGate results on three target files are shown in Supplementary Figure 3.

**Fig. 2:**
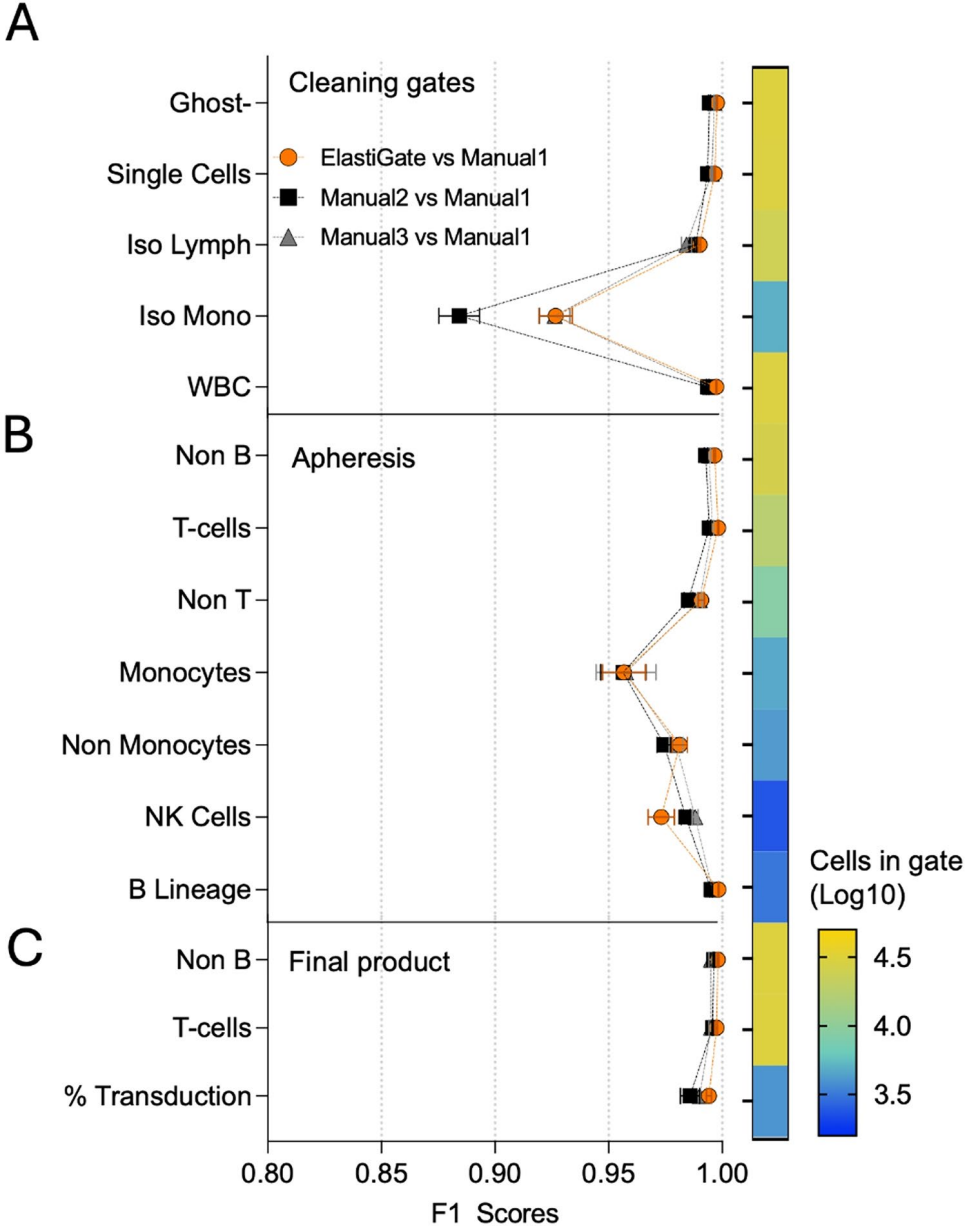
ElastiGate application on a CAR-T cell manufacturing dataset. ElastiGate was used with 3 training files for all apheresis target fcs files and 1 training file for all final product fcs files. The first 5 cleanup gates were applied to all files, and the remaining gates were applied to non-Isotype fcs files. (**A**) The distributions of F1 scores for the first 5 gates over all 71 apheresis and final product target fcs files comparing ElastiGate to gates drawn by three manual expert analysts (Manual 1–3). Values represent the mean and error bars represent standard error of the mean (SEM). The heatmap bar represents number of cells in each gate as a Log10 scale. (**B**) The distributions of F1 scores for the last 7 gates, for 35 apheresis target fcs files (Isotype files are excluded). (**C**) The distributions of F1 scores for 3 gates, for 11 final product target fcs files (Isotype files are excluded). The % Transduction gate was calculated using the “Sample” final product fcs files. Other gates were excluded due to a small cell number, with < 30 cells. WBC: White blood cells.

The results of ElastiGate were compared with those of ground truth manual gates, and multiple manual gating operators were compared with each other (Fig. 2A–C). Here, we report F1 scores for populations for which the ground truth populations all had > 40 cells. Over all gates, the median F1 score for ElastiGate was 0.997, versus 0.993 and 0.995 for the analysts.

#### TIL immunophenotyping

Immunophenotyping is used to identify and characterize immune cell populations in biological samples based on their marker expression and can be particularly complicated in TIL samples due to low cell numbers, reduced separation of cells from debris, and varying cell states. Manual gating of specific subpopulations requires experience and is time consuming due to heterogeneous marker expression and the need to review complex hierarchical gate branches and manually adjust for local variability across multiple samples. To address these challenges, ElastiGate was evaluated on 40 samples stained with a 14-color T-cell panel designed to characterize the CD4^+^ and CD8^+^ immune repertoire of tumor-bearing mice after immunotherapy, including naive/effector/memory states and regulatory T cells.

**Fig. 3:**
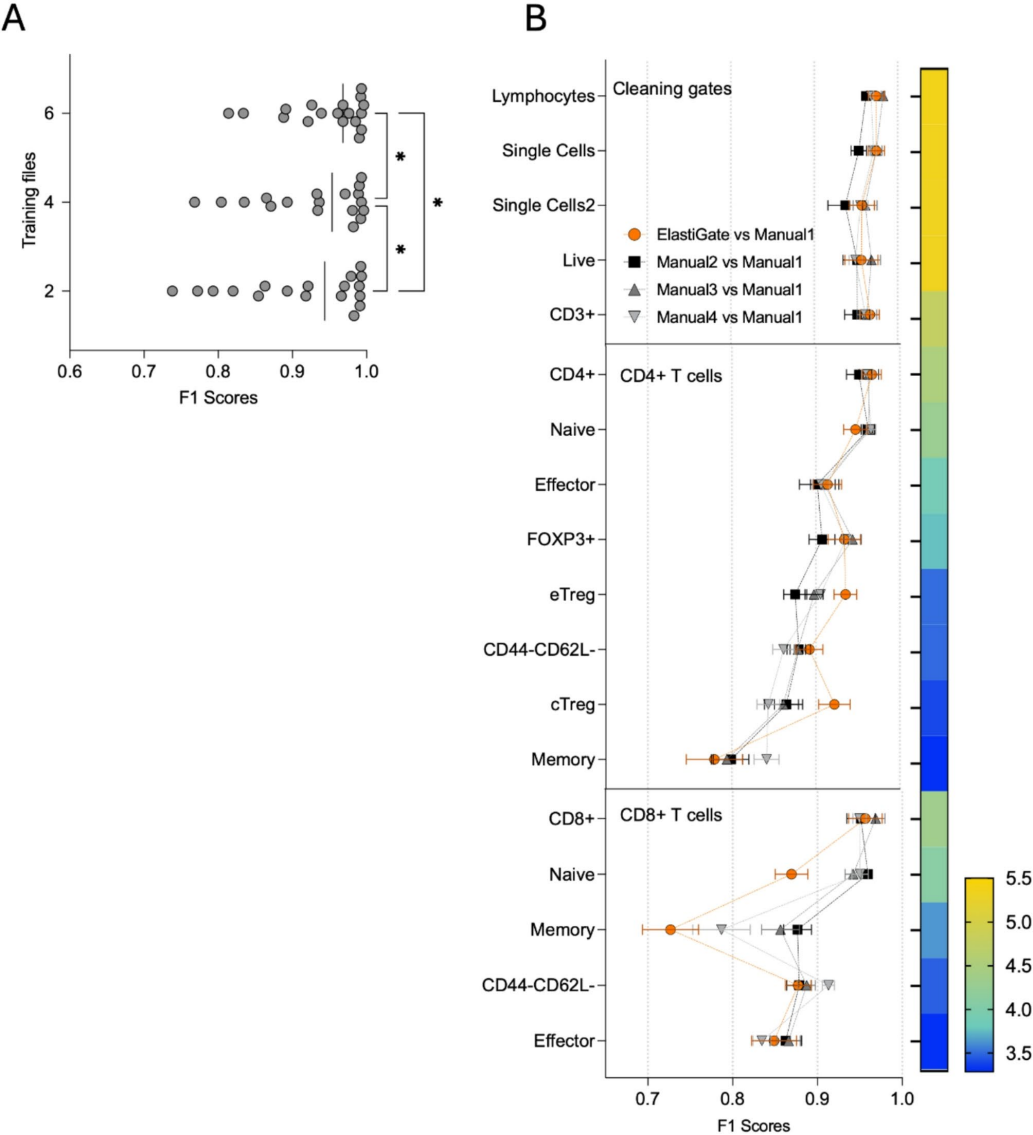
ElastiGate application on a tumor-infiltrating lymphocyte immunophenotyping dataset. (**A**) The mean F1 score for all gates in the tumor-infiltrating lymphocyte immunophenotyping dataset, using 2, 4 and 6 training files. Statistical analyses were performed with non-parametric paired One-Way Anova and the p values were corrected for multiple comparison (Friedman test), *p < 0.05. (**B**) The distributions of F1 scores for each gate over 40 target fcs files in a tumor-infiltrating lymphocyte immunophenotyping dataset comparing ElastiGate to gates drawn by four manual expert analysts (Manual 1–4). Values represent the mean and error bars represent standard error of the mean (SEM). ElastiGate was used with 6 training files. The heatmap bar represents number of cells in each gate as a Log10 scale.

When working with highly complex datasets, we found that a recursive test-and-evaluate approach was useful in enhancing the accuracy of ElastiGate (Fig. 3A). Initially, two sample files were selected as training data. After assessment of the results, two additional files for which the algorithm’s gates needed significant adjustment were added to the training data. The process was repeated, bringing the total number of training files to six, and then ElastiGate was applied to the remaining 34 samples. Four analysts manually gated the 40 samples, and one analysis was randomly selected as ground truth. Example training gates and ElastiGate results on some of the target files are shown in Supplementary Figure 4.

With only two training samples, ElastiGate achieved a minimum median F1 score across all populations of 0.79, with the exception of the memory populations, due to the low number of cells. With six training samples, the minimum median F1 score across all populations increased to 0.8, with an approximately 10% increase in accuracy in some cases (Fig. 3A). ElastiGate and the analysts performed similarly (Fig. 3B), the median F1 scores for all populations being > 0.8. The median F1 scores over all gates for ElastiGate were > 0.935, versus 0.992, 0.943, and 0.943 for the analysts.

#### High-throughput cytotoxicity assay

Flow cytometry cytotoxicity assays are used in screening drug compounds and can be challenging for manual analysis with thousands of samples per day. We tested ElastiGate on 440 samples stained with a seven-color panel to characterize the activation and killing efficiency of T cells against a tumor cell line. The samples were part of a high-throughput plate-based cytotoxicity screening assay with substantial cross-sample variability and populations with low numbers of events, thus the previously explained recursive test-and-evaluate approach was applied. For each drug, samples with the highest and lowest concentrations were selected for the initial training set to capture the maximum and minimum cytotoxicity effect, for a total of 10 samples. After a qualitative data review, five additional samples were added to the training subgroup to fully capture the variability of the data set and further refine the gate placement on the remaining 425 target samples. Five analysts manually gated these 425 samples, and one analysis was randomly selected as ground truth. Example training gates and ElastiGate results on some of the target files are shown in Supplementary Figure 5.

**Fig. 4:**
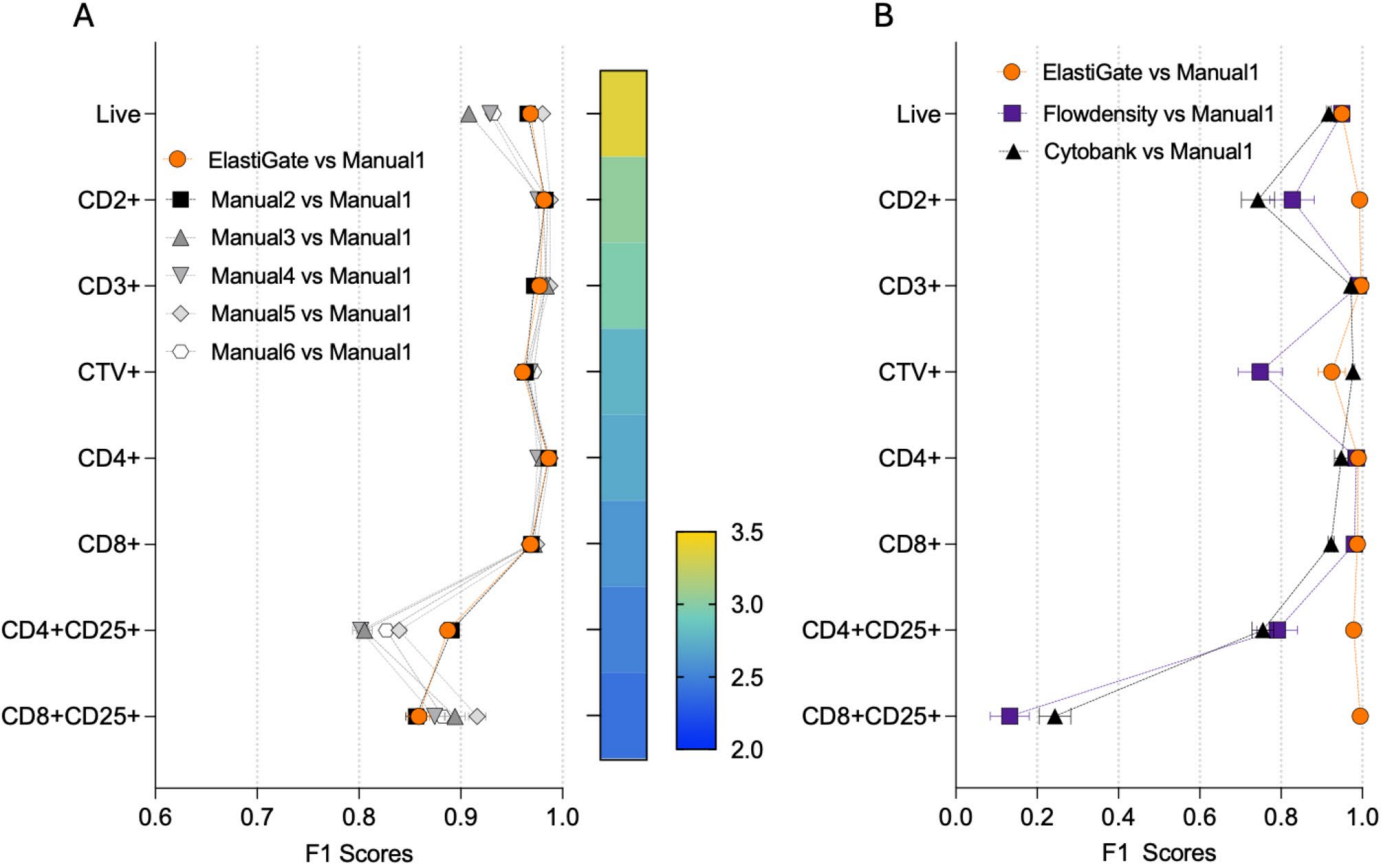
ElastiGate in a high throughput cytotoxicity assay. (**A**) The distributions of F1 scores for each gate over 425 target fcs files in the high throughput cytotoxicity assay comparing ElastiGate to gates drawn by six manual expert analysts (Manual 1–6). ElastiGate was used with 15 training files. Values represent the mean and error bars represent standard error of the mean (SEM). The heatmap bar represents number of cells in each gate as a Log10 scale. (**B**) F1 scores of ElastiGate versus manual expert analyst (orange), FlowDensity versus manual expert analyst (blue), and Cytobank Automated gating versus manual expert (black) from a 38-file subset (one plate) of the dataset. Error bars represent standard error of the mean (SEM). CTV: CellTraceViolet.

F1 scores of manual analysts and ElastiGate were comparable (Fig. 4A), indicating accuracy similar to that of the ground truth. The median of F1 scores over all gates for ElastiGate was 0.969, versus 0.967, 0.968, 0.970, 0.978, and 0.970 for the analysts. ElastiGate F1 scores were at most 0.037 lower than that of the best analyst.

### Comparison of ElastiGate to alternative gating automation tools for 2D plots

To evaluate the gating accuracy of ElastiGate compared with existing technology, a side-by-side analysis was conducted to compare ElastiGate with two other available solutions for automating analysis of two-dimensional plots, flowDensity^9,12^, a well-established density-based method available as R package^12^, and Cytobank Automatic gating, a proprietary method available to Cytobank users^16^. For this analysis, a subset containing 38 samples (38 FCS files) was selected from a single plate from the high-throughput cytotoxicity assay data set described above and used to compare the performance of ElastiGate with flowDensity and Cytobank. For flowDensity, the FCS files were compensated and scaled in FlowJo before being imported as comma-separated value (csv) files into the R library. A specific set of parameters in flowDensity was used to match the corresponding ElastiGate-generated gates as closely as possible and to maximize the F1 scores (see Supplementary Materials). To simplify and standardize the comparison, for each gate, we used as a starting point the parent population derived directly from the manually gated ground truth analysis. This removed the possibility that compounded errors might propagate through a larger gating hierarchy, allowing for a more direct comparison of ElastiGate and flowDensity for each individual gate. Following an initial analysis performed using the default settings, which resulted in a suboptimal gating accuracy, we contacted flowDensity experts to identify critical parameters to improve resolution. The results reported in the current manuscript were obtained using the new set of parameters.

The F1 scores of flowDensity and ElastiGate were comparable for well-defined clustered bimodal distributed populations (e.g., CD3+ gate) but were significantly lower for flowDensity for gates with unclear separation between peaks (Fig. 4B). ElastiGate was able to properly track drastic changes in the live gate to include activated T cells and correctly excluded the dimly positive apoptotic cells, while flowDensity classified the apoptotic cell population as live cells in some cases (Supplementary Figure 6). Similarly, the ElastiGate algorithm captured CD2 downregulation without interfering with the resolution of the Cell Trace Violet+ (CTV+) in proliferating tumor cells, while flowDensity algorithm reverted the horizontal threshold and placed the quad gate (CD2/CTV) on the opposite (incorrect) side of the cluster in some cases, despite the use of optimized input parameters (Supplementary Figure 6). ElastiGate classified the CD2+, CD4+ CD25+, and CD8+ CD25+ gates as expected, with median F1 scores being 0.995, 1.000, and 0.999, respectively. In contrast, flowDensity F1 scores were lower and failed to identify activated CD8+ CD25+ cells. We observed that flowDensity F1 scores were negatively affected by percentage differences between the training and target data. This is reflected by a larger standard error in F1 scores from the ground truth manually gated samples for flowDensity for the CD2+, CD4+ CD25+, and CD8+ CD25+ gates (Fig. 4B). The flowDensity expert developers indicated upon carefully reviewing these results that additional iterative optimization across the parameters by computational experts may further improve the results.

For Cytobank Automatic gating, analysis takes place using a graphical user interface within Cytobank software. The same set of files used for training and inference with flowDensity were used for autogating with Cytobank. Analysis was initially attempted using online tutorials, but due to poor performance on this dataset we contacted technical experts at Cytobank for assistance to help select the ideal training approach given our data. Results were extracted and statistically compared using F1 scores, as shown in Fig. 4B. Results were comparable to flowDensity, with suboptimal gating of CD2+, CD4+ CD25+, and CD8+ CD25+ (but improved performance on CTV+) populations in comparison to other populations and ElastiGate. Overall, in our hands, ElastiGate outperformed flowDensity and Cytobank Automatic gating in terms of both accuracy of results and usability.

### ElastiGate performance with a high-parameter dataset

**Fig. 5:**
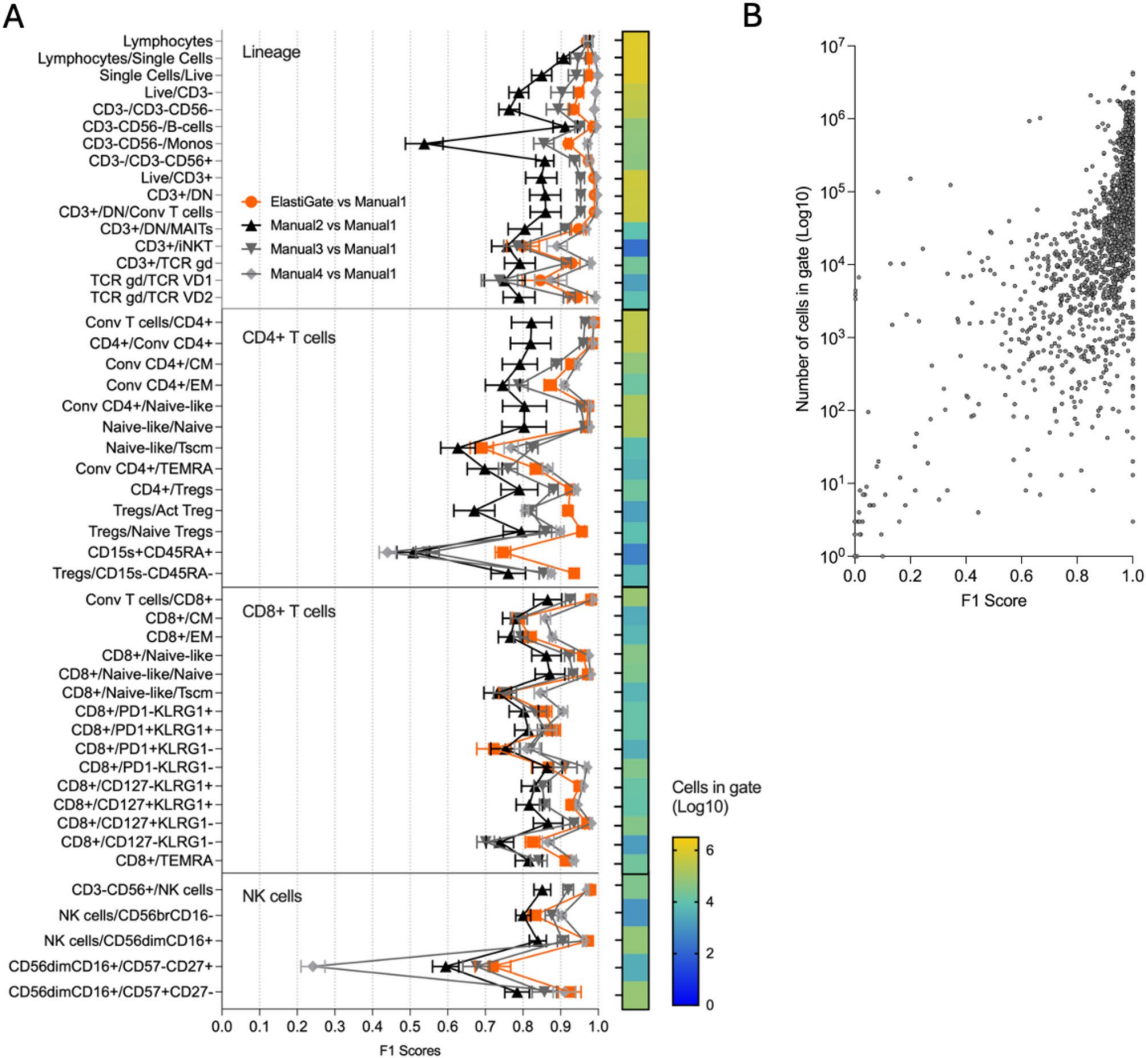
ElastiGate application on 38-color T-cell phenotyping panel. (**A**) The distribution of mean F1 scores comparing ElastiGate to 3 manual analysts for each gate (49 gates in total), with the error bars representing standard error of the mean (SEM). ElastiGate was run on 29 target files using 3 files for training. The heatmap bar represents number of cells in each gate as a Log10 scale. (**B**) The number of cells in each population plotted against it’s ElastiGate F1 score from the 38-color T-cell phenotyping panel.

Spectral flow cytometry and the development of new fluorochromes provide the possibility of analyzing up to 50 markers on a single experiment but poses new challenges for data analysis^17^. This places special burdens on manual analysts due to complex gating strategies with a large number of bivariate plots. To test whether ElastiGate could scale to automate high-parameter data analysis, we selected a 38-color panel developed on a BD FACSymphony™ A5 SE Flow Cytometer primarily to characterize T-cells populations. For this assessment, 32 peripheral blood samples derived from healthy individuals were used. Target files included 25 fresh, 2 frozen, and 2 fixed specimens. We selected 3 freshly isolated PBMC files displaying variation for training ElastiGate and included analysis from 3 manual analysts for comparison. Example training and target gates are shown in Supplementary Figure 7, along with a comparison of how fresh, frozen, and fixed samples performed with the 3 fresh samples selected for training. ElastiGate accuracy, evaluated as F1 score, performed equally or better to manual analysts (Fig. 5). Populations with low F1 scores by ElastiGate also had low F1 scores by manual analysis, driven by the low cell numbers. Overall, these results indicate that ElastiGate can scale to automate the analysis of high-parameter datasets, even with a limited number for training files selected. Of note, it is possible that the analysis of samples from patients and/or drug treatments will require training samples from each group to properly account for biological variability.

### Timing of analysis

ElastiGate markedly reduced the time required for analysis in comparison to manual gating. This held true across all datasets tested (Table 1). The time reduction varied from approximately 3- to approximately 60-fold. On average, about 6 s were required per FCS file, except for the high-parameter dataset with 21 plots that took ~ 60 s per file. In general, computation time correlated with the number of bivariate plots or histograms that were converted to images, resulting in an average of approximately 1–3 s per plot. This average processing time included variation from two laptops, one used for the scatter, bead, monocyte, and CAR-T datasets (Windows 10 running an i5 with a 2.5-GHz processor and 32 GB of RAM), and another used for the TIL, cytotoxicity data, and high-parameter datasets (MacOS Ventura and MacOS Sonoma running a M1 MacBook Pro with 16 GB of RAM). ElastiGate efficiently processed data on standard laptops while substantially reducing the time needed for analysis and reducing subjectivity over manual gating.

**Table 1:** Timing of ElastiGate algorithm and manual gating on all data sets.

Data set	Time to analyze		No. of target files analyzed	No. of plots in gating	Time in seconds for ElastiGate to analyze^a^	
	ElastiGate	Manual gating			File	Plot^b^
Lysed whole-blood	30 s	30 min	30	1	1	1
Quantitation beads	30 s	30 min	20	2	2	1
Monocyte subsets	2 min	2 h	19	7	6	1
Cell therapy QC	15 min	2 h 30 min	72	8	13	2
TIL	6 min	35 min	38	8	9	1
Cytotoxicity assay	45 min	2 h	410	6	7	1
High parameter	30 min	4 h 30 min	29	21	62	3

The average time for ElastiGate analysis was 1 s for a plot and 6 s for a file (excluding the high-parameter dataset, which was 30 s). The overall analysis time needed by ElastiGate was mainly dependent on the number of plots in the gating strategy.

^a^Rounded to nearest whole number.

^b^Plot or histogram.

## Discussion

ElastiGate was developed as a tool to emulate the human process of manually gating flow cytometry data by converting plots to images and adjusting gates to account for local shifts in the data. ElastiGate is released as a FlowJo plugin and in FACSuite with a graphical user interface (UI) with a limited number of intuitive tunable parameters to make the algorithm accessible to scientists with minimal training and no prior coding experience. ElastiGate consistently performed across a variety of flow cytometry assays and instruments in this study and has now been downloaded over 2000 times as a FlowJo plugin. ElastiGate is compatible with multiple gate shapes, including polygon, rectangle, quad, and histogram gates, providing flexibility in the analysis.

ElastiGate was tested on multiple datasets and had accuracy comparable to that of multiple human operators, including sparse multimodally distributed populations, with a median F1 score of 0.95 across all gates with > 5 cells and a median of 0.981 for > 1000 cells, with the exclusion of the high-parameter immunophenotyping assay. The accuracy of ElastiGate was favorable, particularly when taking into account that the operators participating in this study were expert analysts who were aware that their performance was being benchmarked against an automated gating system. Interestingly, in more complex datasets, the contribution of subjective gating choices can introduce inter-operator variability obscuring the biological effect (Figs. 4A and 5A). Compared to manual gating, ElastiGate can reduce analysis time by a factor of 3 to 60 (Table 1), depending on the number of samples and complexity of the gating strategy. Additional time saving can result from reusing a workspace with a curated set of gated training files on new datasets generated using the same assay and instrument settings; the workspace can be also distributed to less experienced analysts to better allocate resources and distribute the workload. No specialized computer hardware is required, and indeed, all the analyses were carried out on standard laptops, owing to the efficient ElastiGate Java back end.

Selection of the training files that reasonably account for any major phenotypic types in the dataset is the most critical step to ensure accuracy. Successful ElastiGate training was achieved with a minimal number of training gated samples, and the number was proportional to the assay complexity and variability of the underlying data. The number of pre-gated training files was 1 for all the development benchmarking data, 3 for the CAR-T cell therapy quality control assay, 6 for the TIL immunophenotyping assay, 15 for high-throughput cytotoxicity screening, and 3 for the high-parameter exploration. Similar results with minimal training files were also shown for assays routinely used in clinical flow cytometry labs^14^. In this study, we found it necessary to expand the training data only for the high-throughput cytotoxicity dataset due to the number of experimental conditions and number of samples. As a general rule, for complex assays with multiple phenotypes, the best results can be obtained by following an iterative training process on a subset of selected samples representative of all the different experimental conditions and expected variability within the data set, as described in the results. We have found 20 to 40 samples to be sufficient for this initial evaluation. It is also essential to note that the training gates should be drawn by a knowledgeable expert: shortcomings in the training gates will be transferred to the automated gates in the test files.

Automated flow cytometry data analysis approaches can be categorized into two main groups: supervised learning, which tackles well-defined problems like identifying specific populations of interest^1,3,5,7,9,10,12,13,18^, and unsupervised learning, which focuses on data exploration and discovery^8,11,19^. ElastiGate falls under the supervised category because it uses training data and training gates. Automated analysis tools using supervised approaches have been developed for pre-specified clinically-oriented assays, with Infinicyt Software and BD FACSCanto Clinical Software among the most popular^2,5,7,20^, however these approaches are not easily generalizable for users to automate analysis of new assays. The open-source nature of flowDensity and similarity to ElastiGate prompted us to compare the two platforms in terms of usability and F1 scores. The F1 scores for flowDensity were comparable for gates containing defined clusters but were significantly lower for gates with no clear bimodal distribution. In contrast to ElastiGate, the gates generated by flowDensity cannot be manually adjusted, and the automated R pipeline requires coding skills to be implemented. To further explore the potential of ElastiGate, we compared it to a proprietary autogating tool with a graphical interface recently added to Cytobank^16^, a cloud-based data analysis platform. The Cytobank autogating approach performed similarly to flowDensity with inaccurate gating placement for populations with continuous distribution and impossibility to manually adjust gates for incorrectly classified populations.

The ElastiGate algorithm is based not on clustering, but on image registration, which allows it to be more generalizable in terms of the shapes and densities of populations that can be gated. There are, however, inherent limitations of this approach. ElastiGate assumes that the relative distance of gate vertices to data densities in the training gates should be preserved when adjusting the gate to new data. In rare cases, and in an attempt to match the provided training gates, this causes gates to not completely or tightly contain a cluster (e.g., gate may overlap or extended outwards from a cluster boundary). Also, ElastiGate assumes all populations are present in the training data. If, for instance, an empty gate is drawn in the training plot, and a new plot contains data in that location, ElastiGate will attempt to move that gate to an empty area of the plot to match the training data. ElastiGate also has some dependency on data density. If areas of a plot are too sparse, ElastiGate may not be able to distinguish between the sparse data and background noise. However, if an entire plot has sparse data, up sampling is performed automatically based on a heuristically chosen threshold of sparseness.

Despite overall good correlation between ElastiGate and manual analysts, lower F1 scores are more common for populations with fewer than ~ 100 cells (Supplementary Figure 8 and Fig. 5B). This is mostly driven by the reduced number of cells to consistently model the shift between training and target data and by the calculation of F1 scores which is very sensitive to individual cell misclassification if the total cell numbers are low. F1 scores are also affected by population distribution and separation between populations. Experts can focus on reviewing these sparsely populated gates to verify proper placement, while saving time by relying on accurate placement for the remaining gates. If the goal is to completely remove the review step for all gates, then additional study should be undertaken for validation. Future work on ElastiGate aims to expand its capabilities and decrease reliance on human intervention. Other limitations of ElastiGate are the lack of support for Boolean gates and FMO workflows. In the current release, users have some options to adjust image normalization, specifically for handling complex plots. Currently, ElastiGate can handle only one or two dimensions at a time, as it is designed to automate conventional manual gating.

In conclusion, ElastiGate is a powerful tool for automating the flow cytometry gating process and has an accuracy comparable to that of manual gating. It works with most gate shapes and population types and can be used with datasets that have substantial experimental or biological variability. Performance can be improved recursively over time by adding more samples to the training group. This tool is ideal for complex data gating strategies and/or high-throughput screening protocols. ElastiGate can be deployed by biologists using widely available software, making gating more objective while retaining accuracy and saving considerable time in analysis.

## Methods

### Algorithm design

The ElastiGate algorithm is composed of three major steps:

1. Conversion of dot plots to pixelated normalized images.
2. Registration of the image of the training plot to the equivalent target plot. The process of image registration is like aligning two pictures, where one picture is distorted to match the other as closely as possible. As a result, a transformation that distorts the training plot to match the target plot is calculated.
3. Application of the calculated transformation to the training plot gates to create gates for the target plots (Supplementary Figure 9).

#### Step 1: Generation of images from dot plots

Conversion of dot plots to images should be conducted on compensated and scaled data with identical scale range to minimize the amount of transformation needed during the image registration step. For each dot plot, a pixel bin map for each dimension is created. The 2D bin counts are normalized and converted into 8-bit gray scales (Supplementary Figure 9A-B, F), which provide adequate display resolution and fast computation time. The normalization method is similar to FlowJo’s method for creating pseudo color plots.

#### Step 2: Image registration

Flow cytometry plots can be thought of as images for which users identify specific subpopulations by drawing gates in 2D space. Each plot or image for a sample can present local changes from an archetype sample due to technical or biological reasons. Gates are typically adjusted manually to account for such local changes. Image registration aims at removing local changes by warping and aligning images, keeping track of the changes that are needed to align individual plots, and then applying the transformation to individual gates.

In image registration, a source image is transformed to align with the target image. In this paper, we refer to the source image as the training image. The goal of image registration is to calculate a function that, when applied to the training image, warps it to look as similar as possible to the target image. Image registration can be rigid or elastic. Rigid transformations are purely global changes to a picture, such as rotation, scaling, and translation. Elastic transformations consist of a collection of localized transformations, so they can adapt to complex changes in different parts of the image (Supplementary Figure 10). ElastiGate uses a modified version of the elastic image registration algorithm bUnwarpJ^21^, which was originally developed for image alignment of microscope images. These modifications are on a branch of the main bUnwarpJ source code, and the modified source code is available at https://github.com/airvine2/bUnwarpJ.

Cubic B-spline interpolation is used by bUnwarpJ to represent the source image and to model the deformation field^22^. B-splines, also known as basis splines, are computationally efficient and can approximate several linear and nonlinear functions^21^. The deformation field is a linear sum of weighted and shifted B-spline functions covering the image area. An example of a deformation field, with the movement shown by arrows, is shown in Supplementary Figure 9D.

The bUnwarpJ algorithm models the images and calculates the deformation field at multiple levels of resolution, which allows consideration of less or more detail^22^. The coefficients of the B-spline model of the image and the deformation are optimized at the coarsest level. Then, the resolution is increased, the previous solution is expanded, and coefficients are recursively optimized (See the Supplementary Materials for more details on B-splines and the deformation field).

When multiple training samples are provided, the ElastiGate algorithm selects the training sample that most closely matches each target sample for each plot. Given a target plot and a collection of training data plots [${I}_{trai{n}_{1}}$, ${I}_{trai{n}_{2}}$, ${I}_{trai{n}_{3}}$, …], it selects the training plot ${I}_{trai{n}_{k}}$ with minimum L_2_ error with respect to the target plot:

$$\underset{k}{\text{min}}\sum_{x,y}{\left({I}_{trai{n}_{k}}(x,y)-{I}_{target}(x,y)\right)}^{2}$$

Once the best training plot is selected, image registration is calculated, and the resulting transformation is applied to the gates as described below.

#### Step 3: Transforming gate vertices

The B-spline transformation generated as a result of the image registration process is finally applied to individual gate vertices of the training data plot to generate gates on the target data plots that reflect such transformation. Several types of gates are supported, including polygon, rectangle, quad, and histogram gates.

#### Histograms

The ElastiGate algorithm also works on histograms. The task of generating images is skipped because histograms are already described by binned data (such as counts by intensity level). Image registration, transformation, and adjustment of gate vertices occurs in one dimension.

#### Quantifying performance

F1 scores were calculated to measure the accuracy of ElastiGate in comparison with manual gating selected as ground truth. To simplify statistical analysis, one of the manually gated datasets (e.g., Manual 1) was selected as ground truth. As a reference point to understand variation between different manual analysts, F1 scores were also calculated between other individuals who performed manual gating (e.g. Manual 2 thru 6, actual number varies between datasets) and ground truth Manual 1. For more details on the F1 score calculation see the Supplementary Materials. For gates with very low cell counts, F1 score does not represent a meaningful accuracy measurement, as a difference of only a few cells can significantly change the value.

#### Statistical analysis of F1 scores

For statistical analysis, the overall median F1 score (across all gated subsets) within one dataset was calculated from the raw F1 scores for each comparison (ElastiGate versus Manual 1, and M2-M6 versus Manual 1), and reported in the main text of the results section. For reporting of F1 score statistics of each gated cell subset within a dataset, the mean and SEM of individual subsets was presented in the main figures.

### Implementation and workflow

#### FlowJo plugin

ElastiGate has been implemented as a FlowJo plugin (see Data Availability), and a dedicated UI was developed to simplify the user experience and apply the algorithm without any coding experience (Supplementary Figure 11). The main FlowJo UI is used to define the gating strategy that will be used for the analysis by drawing gates on the training samples. Then, within the ElastiGate plug-in User Interface (UI), gated training samples and target samples can be selected. ElastiGate can be applied to all or individual gates, and ElastiGate-generated gates can be created underneath existing gates. After completion of the sample and gate selection, the algorithm is executed, and the selected gates will appear on the target samples in the main FlowJo workspace. ElastiGate is separately available with a similar workflow in FACSuite version 1.6.

#### Selection of training data

The selection of the training samples is the most critical step to ensure correct and consistent placement of the gates in the target samples. If the gating strategy is straightforward and samples are homogenous with low variability, a single file can be sufficient to train ElastiGate. More complex cases require more training files. In general, training data should be selected to capture the main phenotypes present in the data set. As a guideline, samples from each median and extreme condition or expected phenotype, including negative and positive controls, unstimulated and stimulated samples, and different drug concentrations, should be included in the training samples.

After ElastiGate is applied, data should be visually reviewed for appropriate gate placement, in particular for gates with poorly defined populations without clear cutoff and/or continuous expression. In the case of suboptimal performance, one or more representative samples (on which ElastiGate results were undesirable) can be manually gated and added to the training set before the algorithm is rerun. In the Results section, we describe how to apply this recursive workflow to increase the accuracy of the median F1 scores by increasing the number of training samples for an immunophenotyping experiment. Our evaluation indicates that a handful of samples are often sufficient to train ElastiGate for an assay, such that it can be applied to future samples processed and acquired using the same parameters and instruments.

#### Options and tuning parameters

The ElastiGate algorithm was designed to expose a minimal number of tuning parameters. “Density level” affects how much the density over the plot is flattened when a density plot is converted to an image. Lower values enhance resolution for sparse populations, whereas higher values filter out sparse areas of the plot. The number of gray levels in the plot image can also be changed to represent more or less detail in density differences. This value may range from 1 to 255. The “interpolate gate vertices” option adds extra gate vertices to the gate at evenly spaced intervals, allowing the gate outline to better follow local changes in the data. Redundant gate vertices are automatically removed after the transformation. The option “preserve rectangles and quads gates” can be disabled to split quadrants into four polygons and rectangles into polygon gates to allow for more freedom of movement and more accurate target gates.

### Flow cytometry data

This study was conducted using pre-existing, de-identified flow cytometry FCS data files. No identifiable information or interventions with human subject or biological samples were needed as part of this data analysis research. Details of flow cytometry methods used to obtain the pre-existing de-identified data files are described in the Supplementary Materials as background information for readers. Availability of flow cytometry data files is also described in the Supplementary Materials. The experimental protocols are aligned with ethics approval from: (1) BD Associate Sample Collection Program (Advarra IRB) for ‘lysed whole blood scatter gating’, ‘monocyte subset analysis’, and ‘high-parameter dataset’, (2) Sanguine (Advarra IRB and WCG IRB) or BioIVT (BIOIVT International IRB) for ‘high throughput cytotoxicity assay’, and (3) Stem Cell Technologies (WCG IRB, Alpha IRB, or NHS Health Research Authority IRB) for ‘cell therapy quality control testing’. For the ‘TIL immunophenotyping’ experiments, mice were obtained from Charles River UK and all animal protocols were approved by the institutional AWERB (Animal Welfare and Ethical Review Board) as well as by the UK Government Home Office under project licence number PP5259481, and all methods are reported in accordance with ARRIVE guidelines (https://arriveguidelines.org). All experiments were performed in accordance with relevant guidelines, including the Declaration of Helsinki, and regulations.

## Electronic supplementary material

Below is the link to the electronic supplementary material.

Supplementary Material 1

## Data Availability

ElastiGate, which is for research use only, not for use in diagnostic or therapeutic procedures, is implemented as a FlowJo plugin, available at https://www.flowjo.com/exchange/#/plugin/profile?id=63. For those without FlowJo, a 30-day free trial is available at https://www.flowjo.com/solutions/flowjo/free-trial. Instructions for plugin installation are at https://docs.flowjo.com/flowjo/plugins-2/installing-plugins. ElastiGate is also available in FACSuite version 1.6. FCS file datasets used in this study are available as described in the Supplementary Materials.
