# Supplementary material for: Automation of flow cytometry data analysis with elastic image registration
Source: Sci Rep. 2025 May 15;15:16949. doi: 10.1038/s41598-025-99118-1 (PMC12081842; doi:10.1038/s41598-025-99118-1)
Supplement: Supplementary file 1 — Supplementary Material 1 [file 41598_2025_99118_MOESM1_ESM.pdf]

Automation of Flow Cytometry Data Analysis with Elastic Image Registration

Allison Irvine†, Mohamed Mahmoud Moustafa†, Sahul Patel, Aniket Patel, Lilja Hardardottir, Francesca Delvecchio, Taylor Foreman, Jean Oak, Scott J. Bornheimer‡, Raffaello Cimbro‡

†‡These authors contributed equally.

Supplementary Materials

Supplementary Figures……………………………………………………………………………..1

Supplementary Text……………………………………………………………………………….15

Supplementary Figures

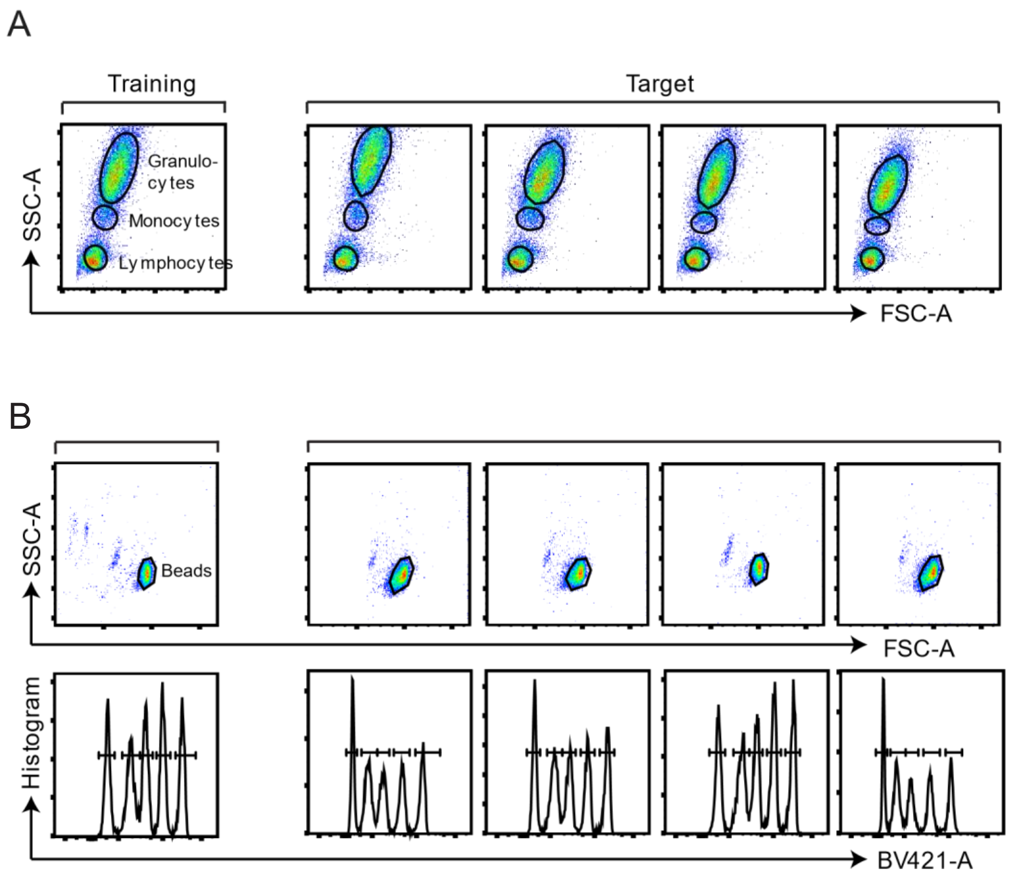

Supplementary Figure 1. Training gates and ElastiGate results on representative target files for (**A**) Lysed whole-blood scatter dataset and (**B**) Multilevel fluorescence quantitation beads data set.

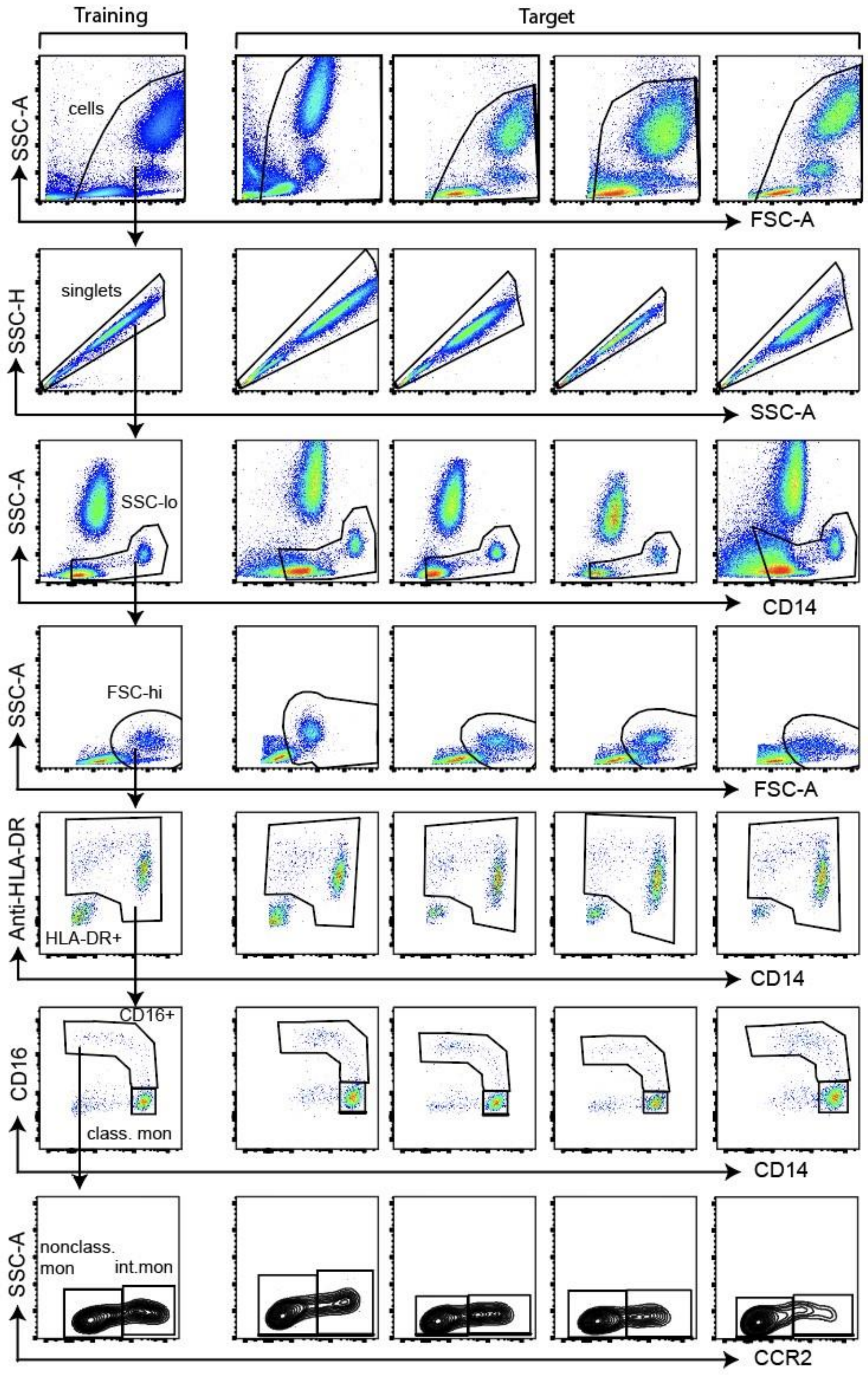
Training
Target
SSC-A
cells
FSC-A
SSC-H
singlets
SSC-A
SSC-A
SSC-lo
CD14
SSC-A
FSC-hi
FSC-A
Anti-HLA-DR
HLA-DR+
CD14
CD16
CD16+
class. mon
CD14
SSC-A
nonclass. mon
int.mon
CCR2

Supplementary Figure 2. Training gates and ElastiGate results on representative target files in the Monoset subset analysis dataset. Class. mon: classical monocytes, nonclass. mon: nonclassical monocytes, int. mon: intermediate monocytes.

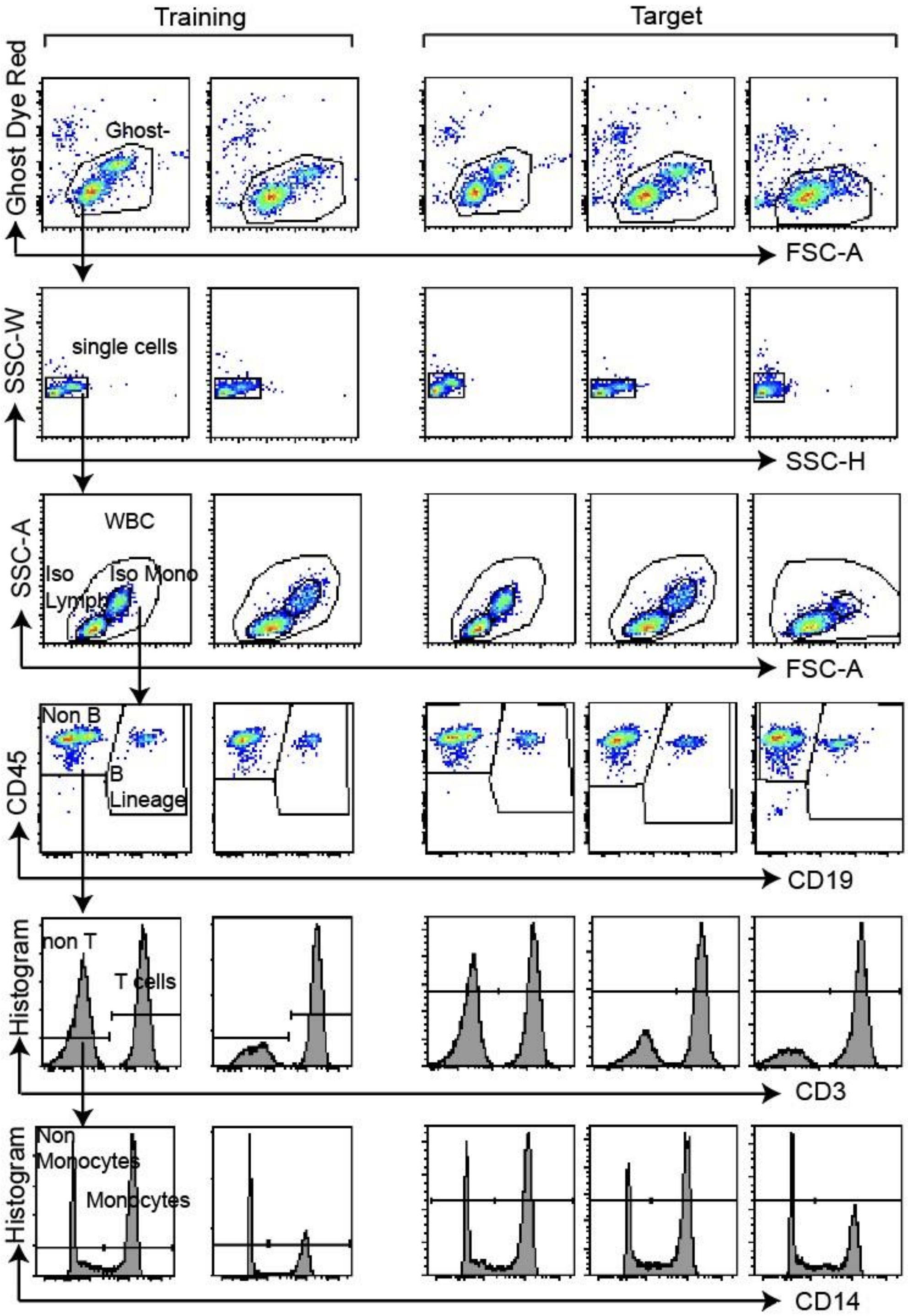

Supplementary Figure 3. Training gates and ElastiGate results on representative target files in the cell therapy quality control testing data set.

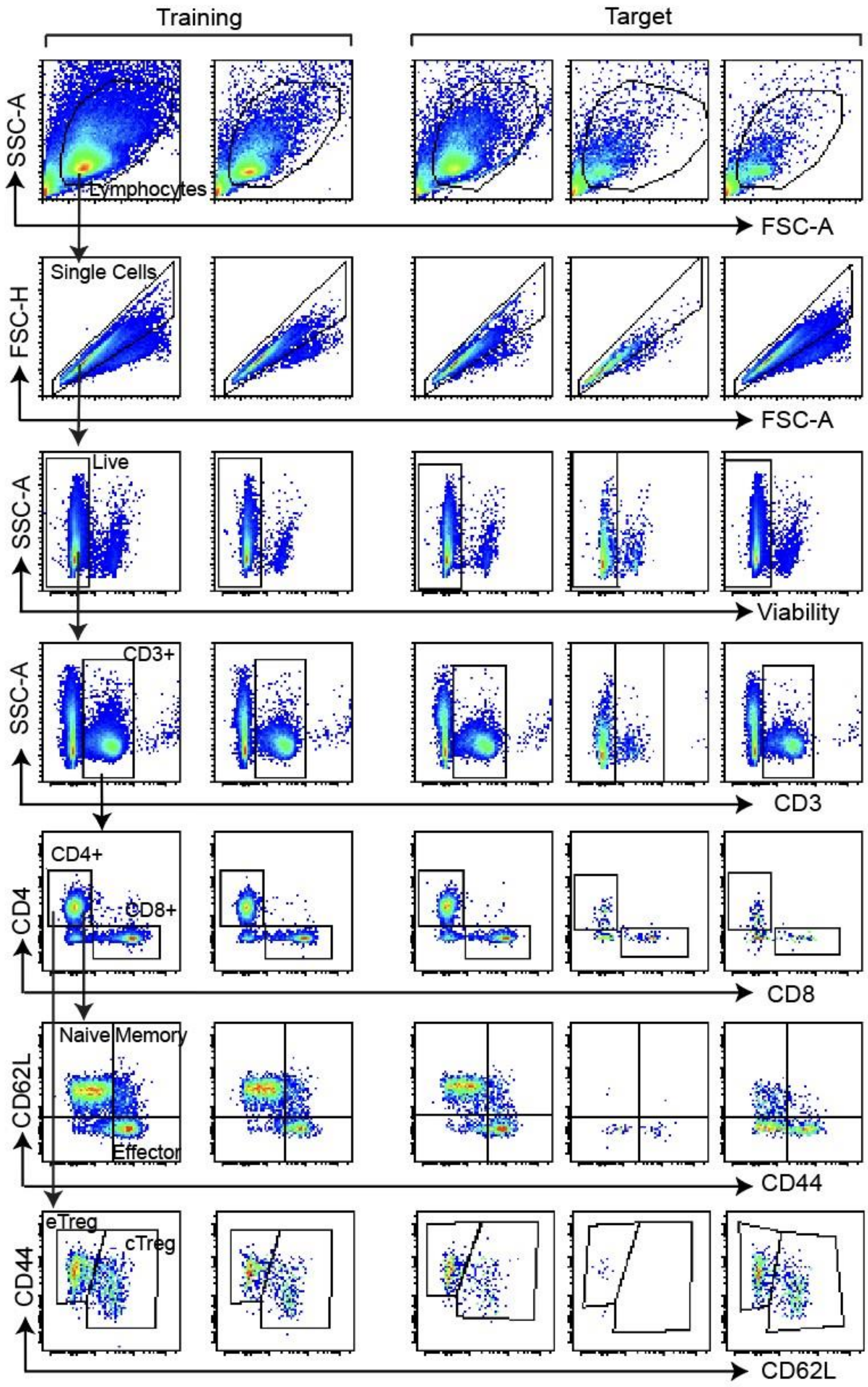
Training
Target
SSC-A
Lymphocytes
FSC-A
FSC-H
Single Cells
FSC-A
SSC-A
Live
Viability
SSC-A
CD3+
CD3
CD4
CD4+
CD8+
CD8
CD62L
Naive
Memory
Effector
CD44
CD44
eTreg
cTreg
CD62L

Supplementary Figure 4. Representative training gates from the six training files used and ElastiGate results on representative target files from the 44 total target files used in the tumor infiltrating lymphocyte immunophenotyping data set.

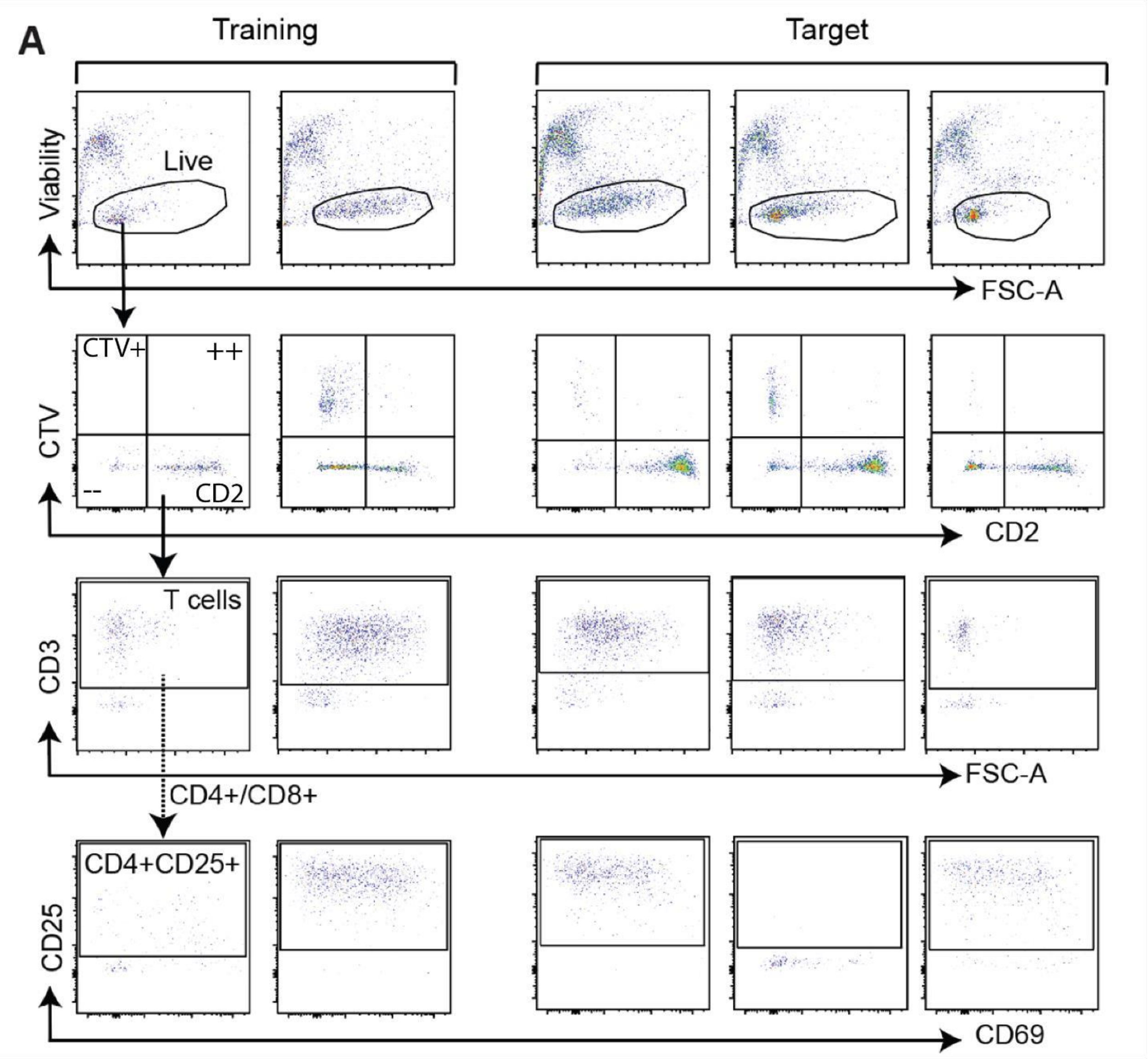

Supplementary Figure 5. Gating strategy from the high throughput cytotoxicity assay. Representative training gates from the 15 training files used and ElastiGate results on representative target files from the 425 total target files. The dotted arrow represents the gating strategy from T cells to CD4+CD25+ gates (the intermediate CD8+ or CD4+ gates are not shown).

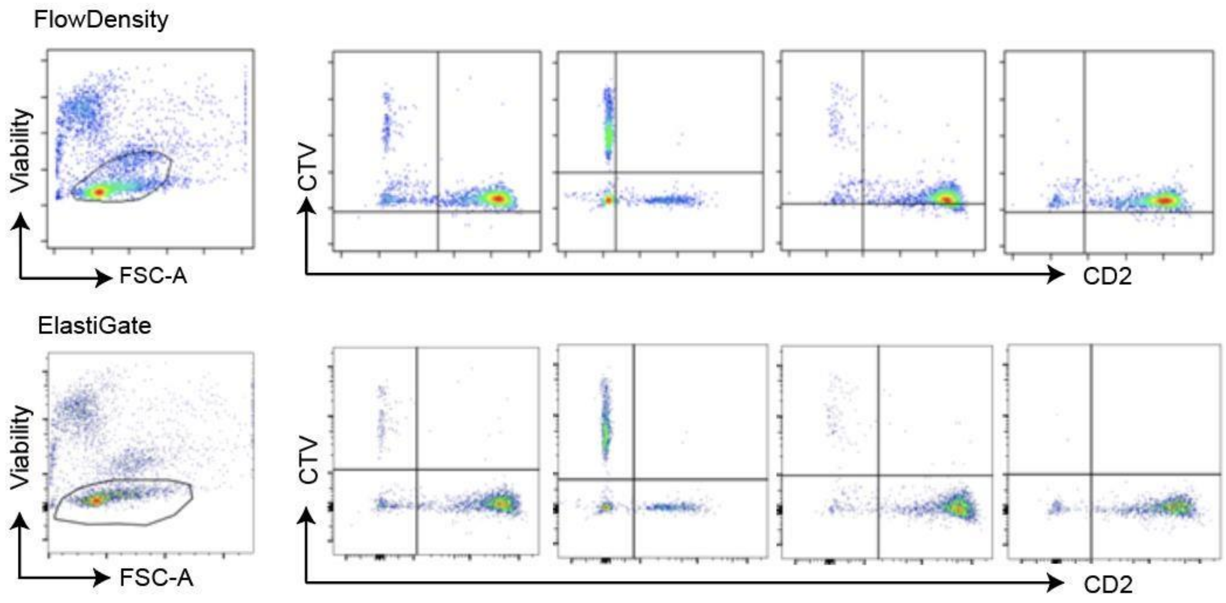

Supplementary Figure 6. Comparison of gates from flowDensity (top row) and ElastiGate (bottom row) algorithms from the high throughput cytotoxicity assay. For visualization of Cytobank Automated Gating, please see Supplementary Figure 12.

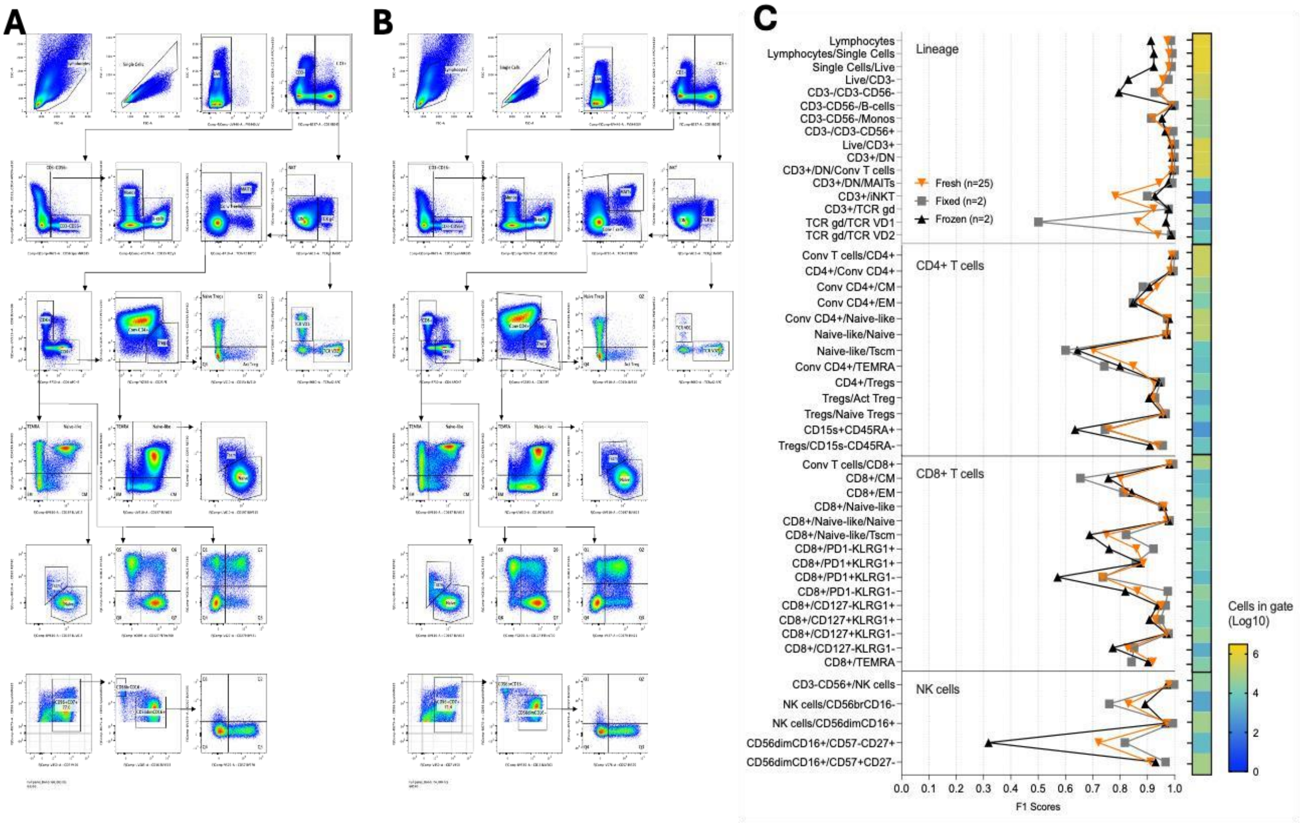

Supplementary Figure 7. Gating strategy from the high parameter dataset. **A)** Representative training gates from one of the three training files shown. **B)** A gating strategy from one of the test files generated by ElastiGate after training. **C)** Comparison of mean F1 score by fresh (n=25), fixed (n=2), or frozen (n=2) sample type. Note the same gating strategy was used for fresh, fixed, and frozen samples.

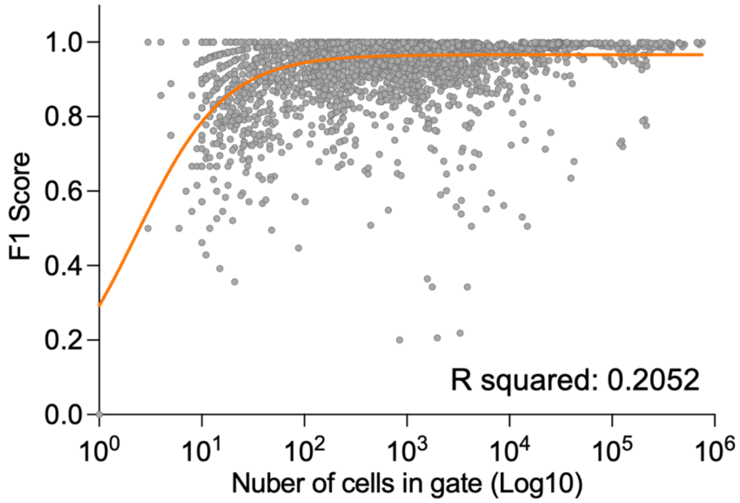

Supplementary Figure 8. Correlation of F1 score and cell counts. Plot includes datapoints from three benchmarking datasets, CAR-T cell manufacturing dataset, tumor-infiltrating lymphocyte immunophenotyping dataset and high throughput cytotoxicity assay, n= 5039. Gates with 10< cells were excluded from the analysis. Orange line indicates nonlinear regression.

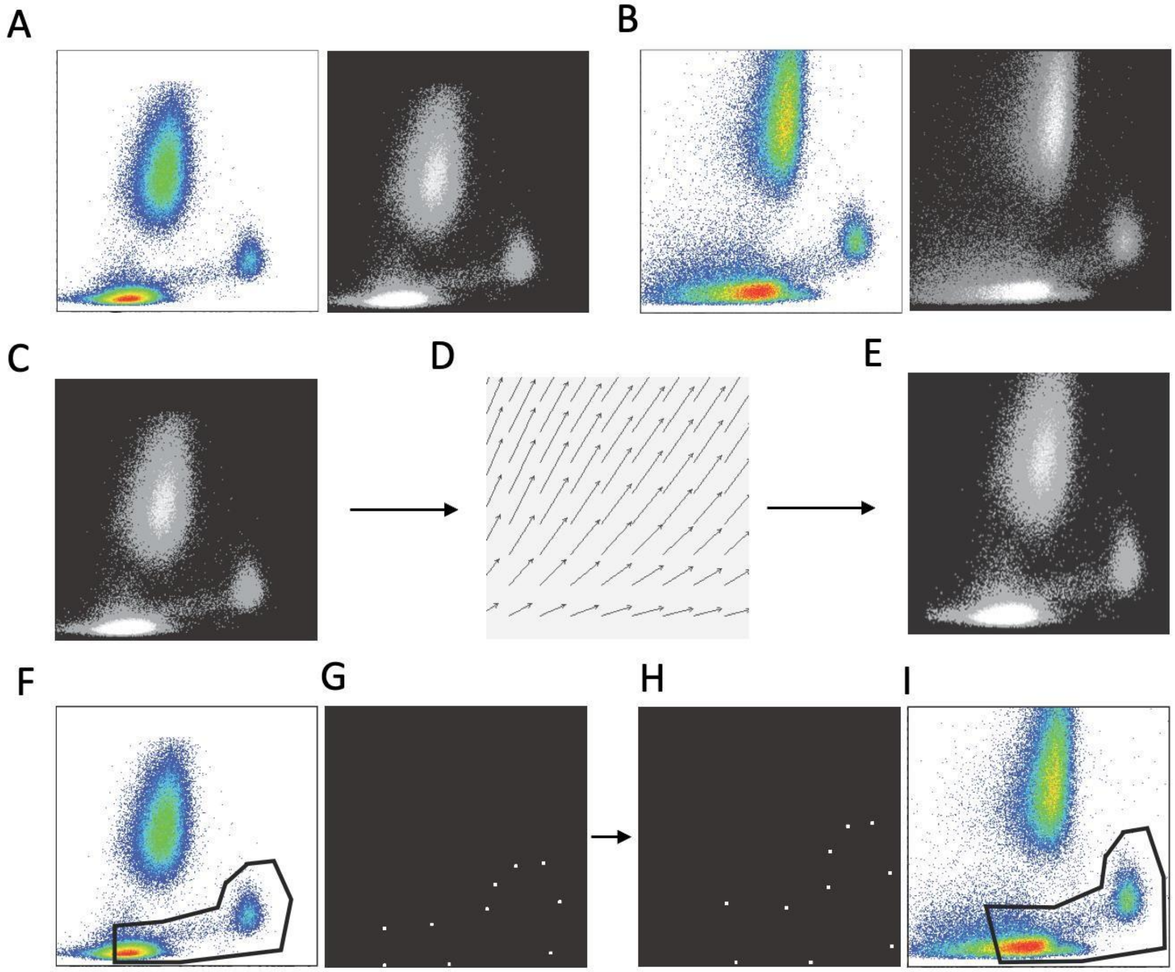

Supplementary Figure 9. A schematic overview of the ElastiGate method.

A) Example training plot (left), and image created from training plot (right). **B)** Target plot (left) and image created from target plot (right). **C)** Training plot image. **D)** Deformation field of the transformation which registers the training plot image to the target plot image. **E)** Registered training plot image. **F)** Original gate vertices of the gate drawn on the training plot. **G)** Gate vertices with the transformation applied to them.

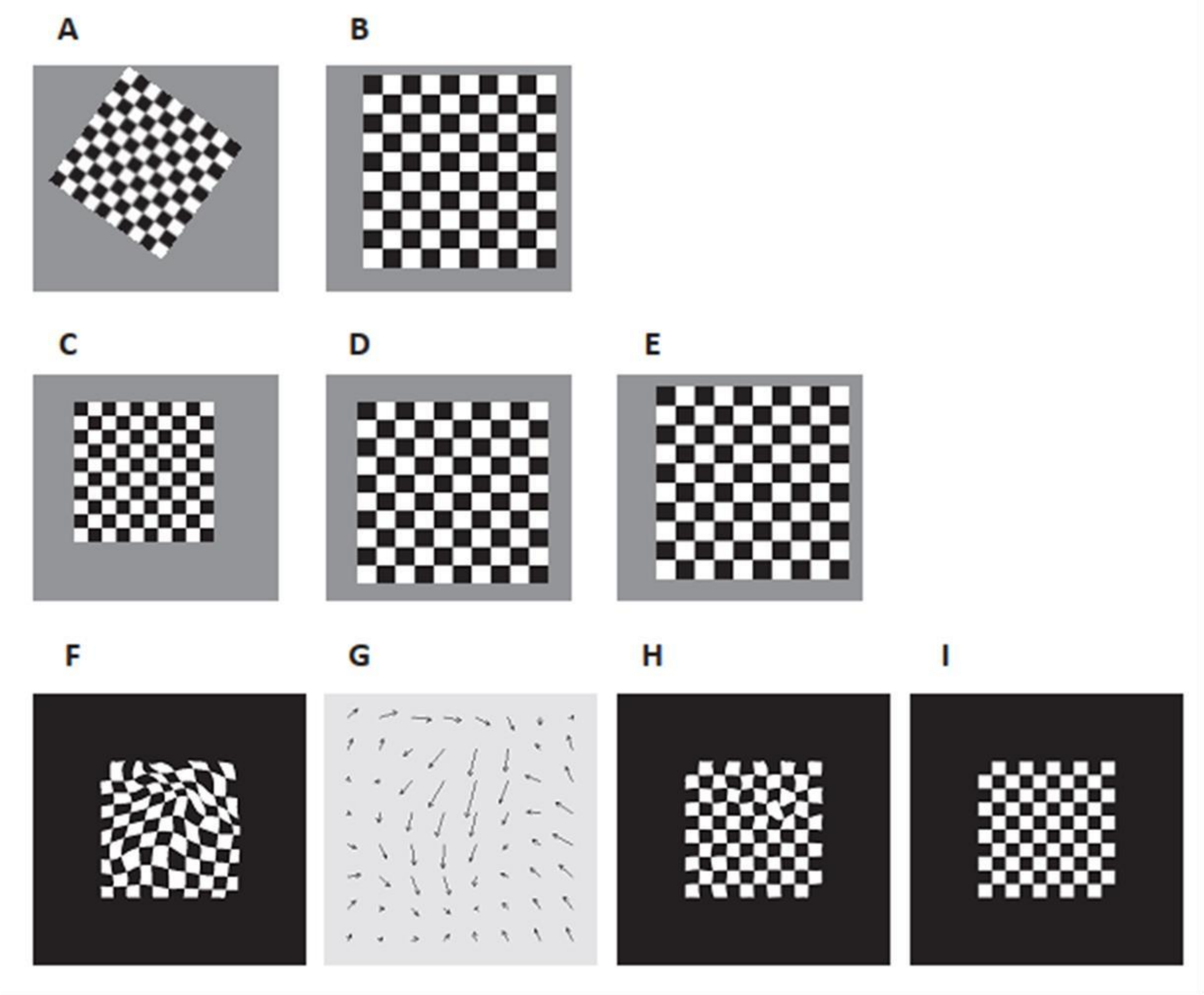

Supplementary Figure 10. Comparing rigid and elastic registration.
A–E) An example of rigid registration. To register the source image **(A)** to the target image **(B)**, the source image is rotated **(C)**, scaled **(D)**, and translated **(E)**. **F-H)** An example of elastic registration. To register the source image **(F)** to the target image **(I)**, the source image undergoes the elastic transformation **(G)** resulting in the registered source image **(H)**. One region of the transformed source image still does not match the target image. This is because the region in the original source image had a sharp irregularity in it and the B-spline functions are smooth, and a limited number of B-spline functions were used to model the image.

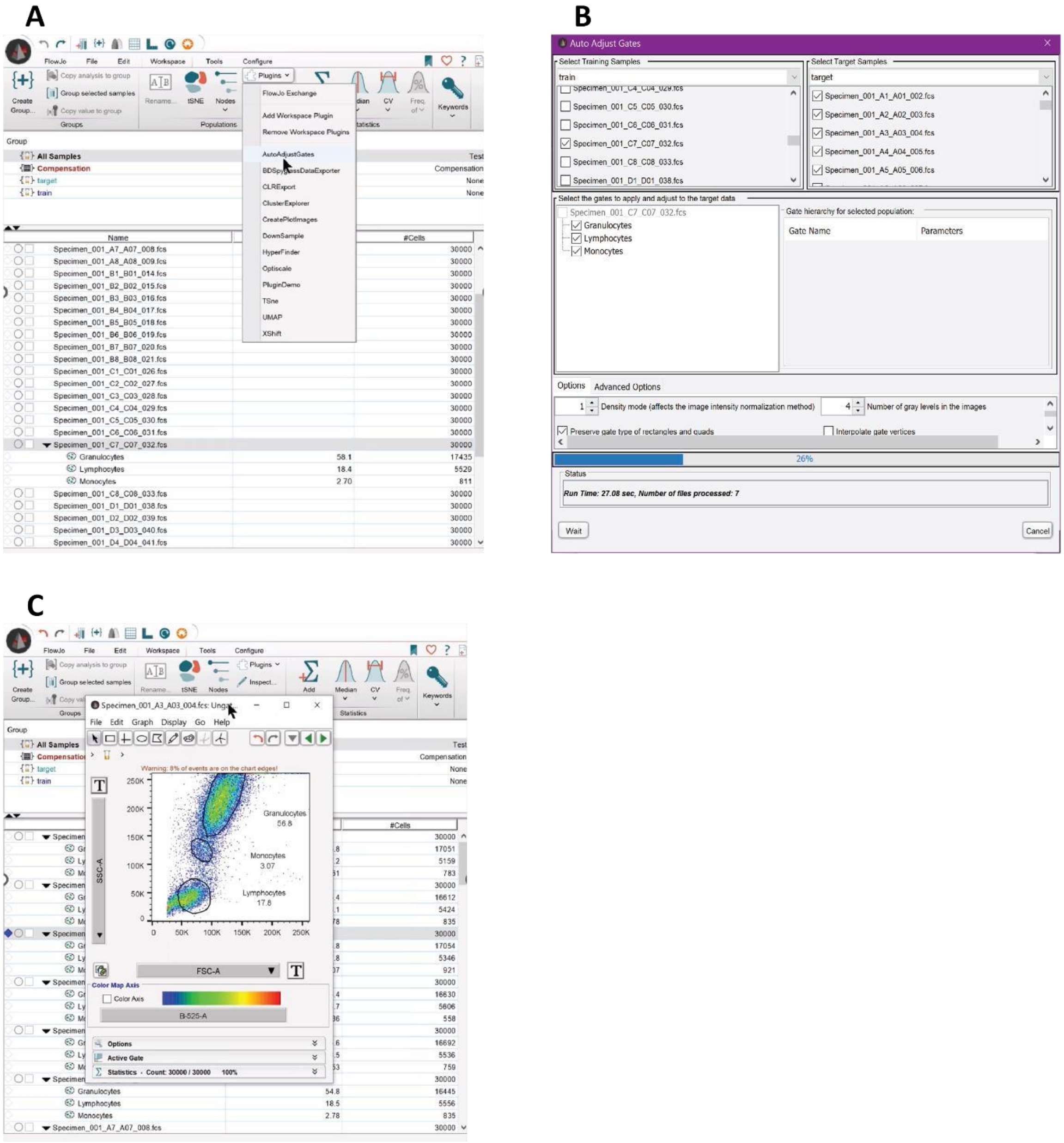

Supplementary Figure 11. Workflow of ElastiGate plugin in FlowJo.

A) An instance of the plugin is created on the gated training sample. **B)** The training samples, target samples, and gates are selected, and then the plugin is started. **C)** After the computation is complete, the User Interface (UI) is closed. At this point all the target samples will have the selected gates drawn on them.

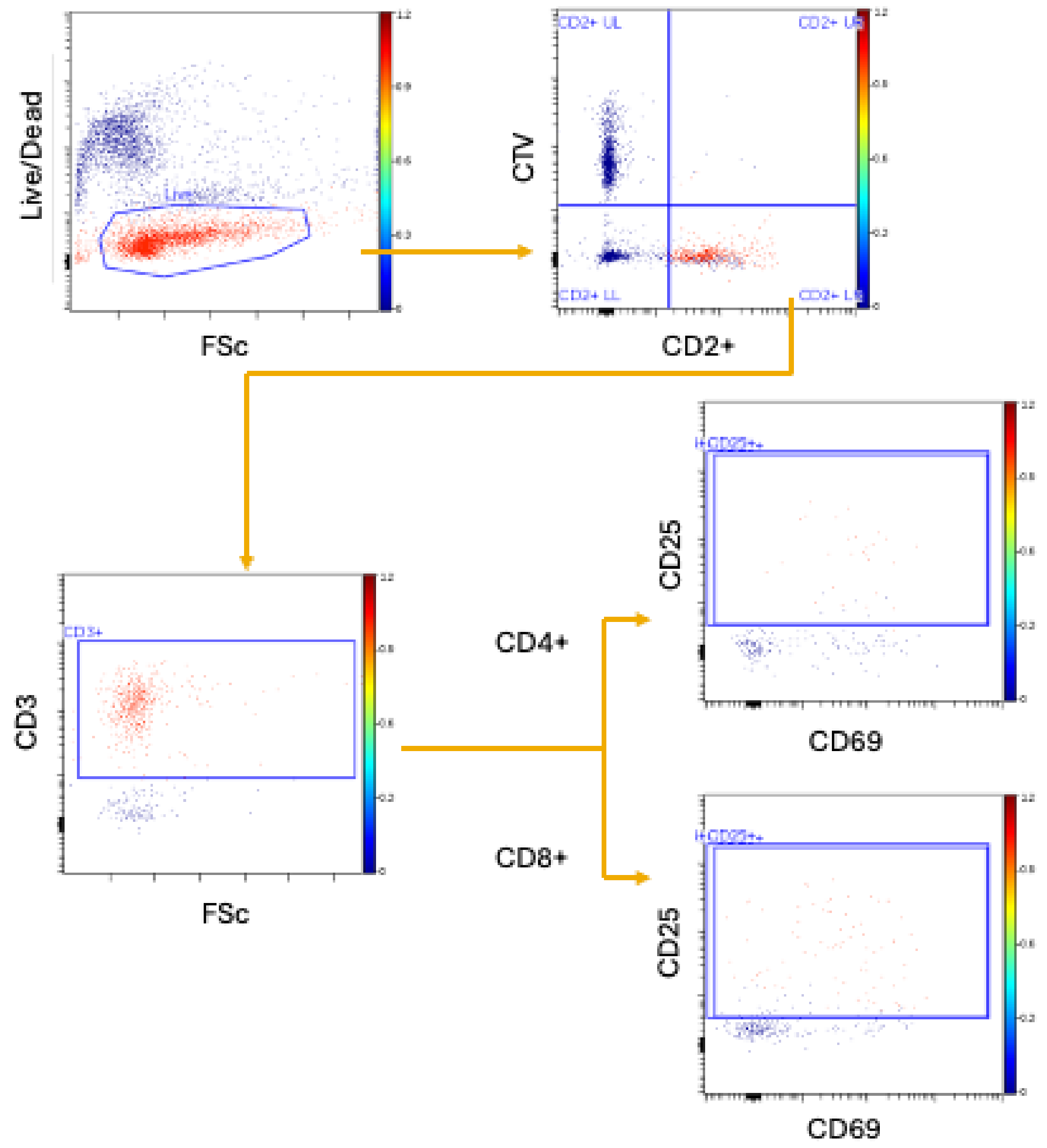

Supplementary Figure 12. Cytobank Autogating visualization.
Overlay of events classified by Cytobank Autogating on gating strategy used for training. Red designates events classified as a gate member by the autogating model.

Supplementary Text

B-splines

B-splines can be used to approximate many different linear and nonlinear functions, are differentiable, and are computationally efficient[1]. B-spline basis functions are described in detail by Racine[2]. They can have different degrees and are defined recursively. Splines are piecewise polynomial functions, and the spots where the pieces connect are called knots. The knots, annotated as t_i, are a non-decreasing sequence of real values, and *i* goes from 0 to N+1, where N>=0. Interior knots are the t_i in between the first and last t_i. Then there is the augmented knot set where $i = 0, \ldots, \mathrm{N} + 2m - 1$, where the lower and upper boundary knots t_0 and t_1 have appended m-1 times. The set of real-valued functions $B_{(i,j)}$ are defined recursively for each augmented knot, and $B_{(i,j)}$ is the i-th B-spline basis function of order j. *n* is the degree of the B-spline basis function and $j = 0, \ldots, n$.

$$B_{(i,0)}(x) = \begin{cases} 1, & t_i \leq x < t_{i+1}, \\ 0 & otherwise \end{cases}$$

$$B_{i,j+1} = w_{i,j+1}(x) B_{i,j}(x) + [1 - w_{i+1,j+1}(x)] B_{i+1,j}(x)$$

$$w_{i,j}(x) = \begin{cases} \frac{x - t_i}{t_{i+j} - t_i}, & t_{i+j} \neq t_i \\ 0 & otherwise \end{cases}$$

$$\sum_i B_{i,n}(x) = 1 \text{ for all } x \text{ between the first and last knot}$$

A B-spline function of degree n, where $(m = n + 1)$ is a parametric curve composed of a linear combination of basis B-splines $B_{(i,n)}(x)$.

$$B(x) = \sum_{i=0}^{N+n} \alpha_i B_{i,n}(x) \,,$$

$$x \in [t_0, t_{N+1}]$$

α_i are the control points, and there are $K = N + m$ of them for a B-spline of order m with N interior knots. In the bUnwarpJ library the source image *I* is represented by a linear combination of degree-3 B-splines[1].

$$I_s(x, y; h) = \sum c_{k,l}\, \beta^3\left(\frac{x}{h} - k\right) \beta^3\left(\frac{y}{h} - l\right)$$

$k,l \in \mathbb{Z}^2$

In this equation, $\beta^3(x)$ is a degree-3 B-spline and $c_{k,l}$ are the coefficients. The value of h and l control the degree of detail of the B-spline model and are a power of 2. Smaller values of h and l result in more detail.

The deformation field $\mathbf{g}(\mathbf{x})$ is a 2-dimensional linear combination of B-splines:

$$\mathbf{g}(\mathbf{x}) = \mathbf{g}(x, y)$$

$$= \left(g_1(x, y), g_2(x, y)\right)$$

$$= \sum \left({}^{1,k,l} \right) \beta^3 \left(- - k \right) \quad c \quad xy \quad \beta_3$$

$$\left(- - l \right)$$

$$2 \quad {}^{2}c\ ,k,l \quad s_x \quad s_y$$

$$k,l \in \mathbb{Z}$$

In this equation, $\beta^3(x)$ is the B-spline of degree 3, $c_{k,l}$ are the coefficients, and s_x and s_y are scalars that control the degree of detail in the deformation field[1]. The coordinates at each position (x, y) are evaluated. The coefficients $c_{1,k,l}$ and $c_{2,k,l}$ are calculated using least-squares fitting. $h = 2^0, 2^1, 2^2, \ldots$ and $l = 2^0, 2^1, 2^2, \ldots$ are parameters controlling amount of detail in the B-spline representation of the source image in the x and y directions, respectively.

The bUnwarpJ algorithm calculates a deformation field by minimizing an energy function. This minimization is performed with a Levenburg-Marquardt algorithm, which is enhanced by estimating the local Hessian of the objective function using the Broyden-Fletcher-Goldfarb-Shanno algorithm[1,3]. The energy function (objective function) being used in our implementation is:

$$E = w_i E_{img} + (w_d E_{div} + w_r E_{rot})$$

This definition omits a few of the terms included in the bUnwarpJ reference paper[1], since they are not being used. The energy function is numerically optimized with respect to the coefficients $c_{k,l}$. E_{img} is the similarity error between the two images. More specifically, it is the sum of squared pixel value differences between the target image and the source image with the current transformation applied to it. E_{div} and E_{rot} are regularization terms based on the divergence and curl of the transformation defined by the coefficients. The w terms are weights on each of the components of the energy function and determine the importance of each of these terms in calculating a transformation.

F1 scores for quantifying performance

For each gate, we examine which cells are inside of the gate and use these numbers to calculate true positives, true negatives, and $F1$ scores.

$$f_1 = 2\left(\frac{precision * recall}{precision + recall}\right)$$

$$precision = \frac{t_p}{(t_p + f_p)}$$

$$recall = \frac{t_p}{(t_p + f_n)}$$

In this equation, t_p is the number of true positives, f_p is the number of false positives, and f_n is the number of false negatives. In cases where the number of cells in the ground truth gate were zero, the F1 score is 1 if the gate being compared also contained 0 cells, and 0 otherwise. In this paper, F1 scores are calculated in comparison to a reference 'ground truth' for each datafile as manually gated by an expert.

Development benchmarking experiments

Lysed whole-blood scatter gating experiments were performed with lysed whole blood from healthy donors acquired on BD LSRFortessa™ X-20 Cell Analyzer. Multilevel fluorescence quantitation bead experiments were obtained with BD custom quantitation beads in BV421 acquired across many days on BD FACSLyric™ flow cytometer. Monocyte subset analysis experiments were performed on lysed whole blood from healthy donors stained with BD Horizon™ Dri Monoset Panel (Table S1) and acquired on BD FACSLyric™ flow cytometer.

Table S1. BD Horizon™ Dri Monoset Panel.

Characteristic	*Analyte*	*Detector*	*Reporter*	*Manufacturer*	*Clone*
monocyte subset	CD16	anti-CD16	FITC	BD	3G8
monocyte subset	HLA-DR	anti-HLA-DR	PE	BD	L243
monocyte marker	CD14	anti-CD14	PerCP	BD	MϕP9
monocyte subset	CD192 (CCR2)	anti-CD192	APC	BD	LS132.1D9

Cell therapy quality control testing

In production of cell therapies, flow cytometry is routinely used for important in-process and final quality control tests. In this example from autologous CAR-T cell therapy manufacturing,

the leukapheresis product from the patient arrives at the manufacturing site and is first tested for the percentage of T and B cells. Further manufacturing occurs including enrichment of T cells and transduction with chimeric antigen receptor against CD19. The final cell product is tested for the percentage of live cells expressing CART-19 as well as impurities. Due to the heterogenous nature of patient-derived cells there is variability in the incoming material and manufacturing result. CAR-T expression in particular is typically a continuous distribution (not a tight peak) presenting challenges for gating. Rigorous gating SOPs must be followed in this environment since the flow cytometry results are an important element in production of a regulated drug product. Many elements of standardized flow cytometry are advisable to include such as using well-maintained and standardized instruments, reagent panels that are dried or lyophilized in pre-titrated, cocktailed form for long term stability, automated sample preparation, operators trained on defined SOPs, and controlling for or removing operator variability in flow cytometry gating. Elements of all of these are included in this particular example and Supplementary Figure 4 and Supplementary Figure 5 provide information on cell populations identified. These data were obtained on BD FACSVerse™ or BD FACSLyric™ flow cytometer. Further details of the specific reagent panel and protocol are proprietary to Novartis.

Tumor infiltrating lymphocyte immunophenotyping

Eight- to ten-week-old female C57BL/6 mice were subcutaneously injected with 100 µL of 5e3 B16 F10 cells in 50% growth factor-reduced matrigel (Corning) and dosed intraperitoneally with either anti-PDL1 antibody (clone 80) mIgG1 (AstraZeneca) or with an isotype control antibody at 10 mg/kg two times per week starting 7 days after tumor cell implantation for a total of six doses.

At day 15 post-implantation, single cell suspensions were obtained by digesting whole tumor at 37°C for 20 min using the Tumor Dissociation Kit (Miltenyi Biotec) and gentleMACS Dissociator (Miltenyi Biotec). A flow cytometry panel was designed to provide a deep dive into intratumoral lymphoid subset immunophenotyping and functional characterization after immunotherapy treatment (Table 1). A 15-color T cell panel defines the activation status of intratumoral CD4+ and CD8+ T cells (naïve, effector memory, central memory) and T regulatory cells (central Tregs and effector Tregs), and the T helper and cytotoxic T cell populations based on the intracellular expression of cytokines. Flow cytometry panel details are shown in Table S2.

The cell suspensions were stained with a fixable viability dye (Thermo Fisher) and blocked with antibodies to CD16/CD32 (eBioscience) before staining with fluorescence-conjugated antibodies in flow cytometry staining buffer (2% bovine serum albumin, 0.1% sodium azide, 2 mM EDTA) with 50% BD Horizon™ Brilliant Stain Buffer (BD Biosciences). FoxP3/Transcription Factor Staining Buffer Set (eBioscience) was used to perform intracellular staining. Data were acquired on a BD FACSymphony™ Flow Cytometer.

Table S2. Tumor infiltrating lymphocyte immunophenotyping panel.

Characteristic	*Analyte*	*Detector*	*Reporter*	*Manufacturer*	*Clone*
Leukocyte marker	CD45	anti-CD45	BUV395	BD	30-F11
Viability	intracellular and extracellular amines	Live/Dead Fixable Blue	Fixable Blue	Invitrogen	N/A
Activation marker	CD44	anti-CD44	BUV737	BD	1M7
Immune checkpoint	PD-1	anti-PD1	BV421	Biolegend	29F.1A12
Exhaustion marker	TIM3	anti-TIM3	BV480	BD	5D12/TIM3
Cytokine signal	IFNg	anti-IFNg	BV605	Biolegend	XMG1.2
Cytotoxic T-cells	CD8a	anti-CD8	BV650	Biolegend	53-6-7
Proliferation marker	Ki67	anti-Ki67	BV711	BD	B56
Lymphocyte homing	CD62L	anti-CD62L	BV786	BD	MEL-14
Cytotoxic effector	Granzyme B	anti-Granzyme B	PE	Biolegend	GB12
Lipid metabolism	neutral lipid droplets	LipidTOX Red	LipidTOX Red	ThermoFisher	N/A
Transcription factor	TCF1/tcf7	anti-TCF1/tcf7	PE/Cy7	Cell Signal	C63D9
Treg marker	Foxp3	anti-Foxp3	APC	Ebioscience	FJK-16S
Helper T-cells	CD4	anti-CD4	AF700	Biolegend	RM4.5
T-cell receptor	CD3	anti-CD3	APC-Cy7	BD	145-2C11

High-throughput cytotoxicity assay

CTV-labelled OCILy18 (Target) were mixed with PBMC from healthy donor (Effector) at a 5:1 E:T ratio in U-bottom 96 well plates and cultured with the indicated doses of drugs. After 3 days, cells were stained with CD2 (Clone RPA-2.10), CD3 (Clone SP34-2), CD4 (Clone OKT4), CD8, CD25 (Clone 2A3) and CD69 (Clone FN50) to determine T cell activation status (panel summarized in Table S3). Cytotoxicity activity was calculated based on the fraction of CTV+ Target cells in each well with T cell engagers compared to control wells without drugs. Data were acquired on a BD FACSymphony^{TM} Flow Cytometer.

Table S3. High-throughput cytotoxicity assay panel.

Characteristic	*Analyte*	*Detector*	*Reporter*	*Manufacturer*	*Clone*
Pan T-cell marker	CD2	anti-CD2	APC-H7	BD	RPA-2.10
T-cell receptor	CD3	anti-CD3	BV605	BD	SP34-2
Helper T-cells	CD4	anti-CD4	PE-Cy7	Biolegend	OKT4
Cytotoxic T-cells	CD8	anti-CD8	confidential	confidential	confidential
T-cell activation	CD25	anti-CD25	BUV737	BD	2A3
T-cell activation	CD69	anti-CD69	PE	Biolegend	FN50
Viability	intracellular and extracellular amines	Live/Dead Fixable Green	FIxable Green	Thermofisher	N/A
Proliferation tracker	intracellular amines	CellTraceViolet	CellTraceViolet	Thermofisher	N/A

Comparison of ElastiGate to alternative gating automation tools for 2D plots

To use flowDensity, we tuned parameters to model each gate as closely as possible resulting in one customized flowDensity function call for each gate. There is no training data in flowDensity, only programmatic definitions of where the gate boundaries are, for each gate. To see how the parameters affect the accuracy of results, we tried three different parameter settings for each gate. We then calculated F1 scores of the results for each of these, and for each gate used the parameter settings which resulted in the highest F1 score for our comparative study. The parameter settings used for FlowDensity were as follows:

```
#"Live"

f1 = function(x){

        fdResult = flowDensity(obj=x,channels=c(1:2),position=c(T, F), percentile=c(0.3,
NA),use.percentile=c(T, F), ellip.gate=TRUE,scale=0.85)

        return(fdResult@index)

}


#"CD2+"

f4 = function(x){

        threshX <- deGate(obj = x,channel = 1, upper=F)

threshY <- deGate(obj = x,channel = 2, upper=T)

return(which((x[,1]>threshX) & (x[,2]<=threshY)))

}


# "CTV+"

f5 = function(x){

        threshX <- deGate(obj = x,channel = 1, upper=F)

threshY <- deGate(obj = x,channel = 2, upper=T)

return(which((x[,1]<=threshX) & (x[,2]>threshY)))

}
```

```
# "CD3+"

f6 = function(x){

            threshY <- deGate(obj = x,channel = 2,use.upper=F,upper=F,alpha = 0.1)

return(which((x[,2]>threshY)))

}


# "CD4+"            f7

= function(x){


            threshX <- deGate(obj = x,channel = 1, upper=T)

threshY <- deGate(obj = x,channel = 2, upper=T)

 fdResult = flowDensity(obj=x, channels=c(1:2),position=c(FALSE, TRUE), gates =
c(threshX,threshY))

            return(fdResult@index)

}


# "CD8+"

f8 = function(x){

            threshY <- deGate(obj = x,channel = 2,upper=T)

return(which(x[,2]<=threshY))

}


# "CD4+/CD25+"

f9 = function(x){

            threshY <- deGate(obj = x,channel = 2,use.percentile = T, percentile = 0.09,
use.upper=T,upper=T)            return(which(x[,2]>threshY))

}
```

```
# "CD8+/CD25+"                              f10
= function(x){


        threshY <- deGate(obj = x,channel = 2,use.upper=T,upper=T,percentile = 0.1)
return(which(x[,2]>threshY))
}
```

For our comparison with Cytobank's Automatic gating, the same training and test samples were used as for the flowDensity comparison. The gating was recreated within Cytobank's platform and used for training of the model. The event classification was exported and used to evaluate performance against ElastiGate.

To evaluate the accuracy of each gate independently of error introduced by applying the algorithm to the parent gates, we calculated each automatic gate on the manually gated parent population, for these comparisons only. Note that, for the ElastiGate method discussed in this paper, we used the parent population created by the automatically adjusted gates. This means that as one goes further down the gating hierarchy, errors introduced by the automatically adjusted parent gate are compounded with any errors introduced by the next child gate. Since the error was generally low for our ElastiGate algorithm, we did not feel the need to remove these compounded errors before reporting results in the rest of the paper.

High-parameter dataset

Peripheral blood mononuclear cells (PBMCs) were obtained from healthy volunteers as per institute guidelines on specimen collection. PBMCs were separated from the whole blood following Ficoll-Paque density gradient centrifugation. For immunofluorescence staining, two different batches of antibody cocktails were made. For the first batch, designated as "preincubation cocktail" antibodies directed to chemokine receptors (CXCRs), CD94, TCR-GD and fixable viable stain dye (FVS440UV) were included. This was done to avoid either antibody competition or allow CXCR receptor occupation before targeting remaining cell surface markers in the panel. Prior to the actual experiment, all fluorescent antibodies and live stain dye were titrated to optimize performance in the multicolor panel. To avoid non-specific Fc receptor mediated fluorescent antibody binding, human Fc Block (BD Fc Block Cat.564220) was used. The pre-incubation cocktail recipe includes BSB+ (BD Cat No.566385), pre-defined antibody mixture and buffer (1X PBS). Final volume was adjusted so that total staining volume does not exceed 150 □L per test. Pre-incubation cocktail was added to the cell pellet ($5x10^6$) in a 96-well microtiter

plate and incubated at 37ºC, for 10min in dark. In parallel, the remaining antibody cocktail was prepared. After, the completion of pre-incubation steps, the second antibody cocktail was added, and the plate was incubated at room temperature in the dark for 30 minutes. For single color stains, a total of one million PBMCs were used for each fluorochrome antibody combination and similar incubation steps as done for full panel was followed. For markers whose surface expression was inherently low, we used corresponding anti-human CD4 bound fluorochromes as single-color compensation control. A set of compensation beads, anti-mouse Ig kappa (BD cat. 562843) were stained as additional compensation controls and used in the analysis when needed. Unless specified otherwise, similar antibody volumes were used for bead comp as have been tested for panel. Manufacturer specified procedures were followed for staining bead controls (30min RT with two wash cycles, without any preincubation of antibody step). After the incubation steps were over, the plates were centrifuged briefly (1400 rpm X 5 min), decanted thoroughly, followed by wash cycles (1_X FACS wash buffer (1400 rpm $_X$ 5 min), 3_X times). After each wash, extra liquid in the wells was removed carefully by gently tapping the plate onto soft absorbents. After completion of wash steps, stained samples were resuspended in 1X FACS wash buffer and kept on ice and protected from light until acquisition. Single color stains were acquired for spectral unmixing after confirming correct expression of individual markers. Samples stained with the complete multicolor panel (details available upon request) were acquired next along with single stained controls. All acquisition was performed at a defined acquisition (events per second) rate on a BD FACSymphony™ A5 SE Cell Analyzer. A total of 1 million events were acquired per fully stained sample and at least 10K events were acquired for single color controls. Unmixing was then performed in FlowJo using acquired single stained controls for each run.

FCS file data availability

FCS file datasets from most analyses in this study are available for download. This includes all files from the analyses of scatter gate, multilevel fluorescence quantitation beads, monocyte subsets, and TIL immunophenotyping. For the high throughput cytotoxicity assay, due to the extremely large number of files, only FCS files from one plate are provided, which were also used for the comparison to flowDensity and Cytobank Automated Gating. For the cell therapy quality control dataset, these files could not be made publicly available due to confidential information of Novartis; interested parties can contact the authors. For the high parameter dataset, due to ongoing development and improvement of the panel the data contributors request that interested parties contact the authors.

Lysed whole blood scatter gating: https://figshare.com/s/1df6921a1a01b31ac0a9

Multilevel fluorescence quantitation beads: https://figshare.com/s/359157a95ce1c76a4891

Monocyte subset analysis: https://figshare.com/s/094d09fb2721958742a9

TIL immunophenotyping: https://figshare.com/s/8d6079bb681fe99013c9

High throughput cytotoxicity assay: https://figshare.com/s/bc88e5969e37b9426284

References

1. C. O. S. Sorzano, P. Thevenaz, & M. Unser. Elastic registration of biological images using vector-spline regularization. IEEE Trans. Biomed. Eng. 52, 652–663 (2005).

2. Racine, Jeffrey S. n.d. “A Primer on Regression Splines.” https://cran.rproject.org/web/packages/crs/vignettes/spline_primer.pdf

3. Arganda-Carreras, I. et al. Consistent and Elastic Registration of Histological Sections Using Vector-Spline Regularization. Lecture Notes in Computer Science 4241, 85-95 (2006).
